# Reporting on invasive lobular breast cancer in clinical trials: a systematic review

**DOI:** 10.1038/s41523-024-00627-5

**Published:** 2024-03-20

**Authors:** Karen Van Baelen, Josephine Van Cauwenberge, Marion Maetens, Gabriela Beck, Ann Camden, Megan-Claire Chase, Valerie Fraser, Siobhan Freeney, Laurie Hutcheson, Julia K. Levine, Tone Lien, Rian Terveer, Claire Turner, Elzbieta Senkus, Rachel C. Jankowitz, Vincent Vandecaveye, Giuseppe Floris, Patrick Neven, Hans Wildiers, Elinor Sawyer, Anne Vincent-Salomon, Patrick W. B. Derksen, Christine Desmedt

**Affiliations:** 1 Laboratory for Translational Breast Cancer Research, Department of Oncology, KU Leuven, Leuven, Belgium; 2 Department of Gynecological Oncology, University Hospitals Leuven, Leuven, Belgium; 3 European Lobular Breast Cancer Consortium, Utrecht, the Netherlands; 4 Lobular Breast Cancer Alliance inc., Plymouth, MA USA; 5 Lobular Ireland, Dublin, Ireland; 6 Borstkankervereniging Nederland, Utrecht, the Netherlands; 7 Lobular Breast Cancer UK, Manchester, UK; 8 Department of Oncology and Radiotherapy, Medical University of Gdańsk, Gdańsk, Poland; 9 Division of Hematology/Oncology, Abramsom Cancer Center, University of Pennsylvania, Philadelphia, USA; 10 Department of Radiology, University Hospitals Leuven, Leuven, Belgium; 11 Division of Translational MRI, Department of Imaging and Pathology, KU Leuven, Leuven, Belgium; 12 Department of Pathology, University Hospitals Leuven, Leuven, Belgium; 13 Department of General Medical Oncology, University Hospitals Leuven, Leuven, Belgium; 14 School of Cancer and Pharmaceutical Sciences, Faculty of Life Sciences and Medicine, Guy’s Cancer Centre, King’s College London, London, UK; 15 Department of Pathology, Institut Curie, Paris Sciences Lettres University, Paris, France; 16 Department of Pathology, University Medical Center Utrecht, Utrecht, the Netherlands

**Keywords:** Breast cancer, Drug development

## Abstract

Invasive lobular breast cancer (ILC) differs from invasive breast cancer of no special type in many ways. Evidence on treatment efficacy for ILC is, however, lacking. We studied the degree of documentation and representation of ILC in phase III/IV clinical trials for novel breast cancer treatments. Trials were identified on Pubmed and clinicaltrials.gov. Inclusion/exclusion criteria were reviewed for requirements on histological subtype and tumor measurability. Documentation of ILC was assessed and ILC inclusion rate, central pathology and subgroup analyses were evaluated. Inclusion restrictions concerning tumor measurability were found in 39/93 manuscripts. Inclusion rates for ILC were documented in 13/93 manuscripts and varied between 2.0 and 26.0%. No central pathology for ILC was reported and 3/13 manuscripts had ILC sub-analyses. ILC is largely disregarded in most trials with poor representation and documentation. The current inclusion criteria using RECIST v1.1, fall short in recognizing the unique non-measurable metastatic infiltration of ILC.

## Introduction

Invasive lobular breast cancer (ILC) is the second most common histological type of breast cancer (BC) and differentiates itself from invasive breast cancer of no special type (IBC-NST) at the clinical, histological and molecular level^1–5^. The majority of ILC belong to a luminal surrogate intrinsic subtype since >90% are hormone sensitive and lack *HER2* amplification^6^. The pathological diagnosis of ILC relies, according to the World Health Organization’s (WHO) Classification of Breast Tumors, on the non-cohesive nature and single file or targetoid pattern of tumor cells observed on routine histological examination^7^. This definition seems however insufficient since several retrospective analyses revealed only a limited agreement between local and central pathology in diagnosing ILC^8,9^.

In current clinical practice, implications of ILC histology on treatment choices are limited^5^. Evidence on the efficacy of novel BC treatments in patients with ILC is often missing^5,10^. Differential responses to the long-used pillars of BC treatment, endocrine therapy and chemotherapy, have been demonstrated in early stage between patients with ILC and patients with IBC-NST^11–13^. The use of (neo-)adjuvant chemotherapy regimens is believed to be less beneficial in patients with ILC compared to patients with IBC-NST, even when correcting for surrogate intrinsic subtypes^12,13^. Data from the BIG 1-98 trial suggested improved outcomes for the adjuvant use of aromatase inhibitors (AI) over tamoxifen only in patients with ILC^11^. Recent data concluded that the use of AI is also preferred in patients with IBC-NST however the magnitude of outcome improvement might still be greater for patients with ILC^14^.

Survival of patients with BC has steadily improved with the introduction of several novel BC treatments^15^. The landscape of BC treatment has broadened with the development of CDK4/6 inhibitors, oral selective estrogen receptor degrader (SERDs), immune checkpoint inhibitors (ICI), antibody-drug conjugates (ADCs) and other novel drug classes. While pure (i.e. not mixed with IBC-NST) lobular histology is found in approximately 15% of all BC^16^, the efficacy of these novel drugs for patients with ILC is currently understudied^5^. Abel and colleagues provided some first quantitative insights on this topic by retrospectively assessing the enrollment of patients with ILC in clinical drug trials conducted in their institution^17^. They reported that while 17.9% of all stage IV BC treated in that center had ILC, only 9.2% of all patients with BC included in stage IV clinical drug trials were patients with ILC.

One hypothesis to explain lower enrollment of patients with ILC is that the Response Evaluation Criteria in Solid Tumors version 1.1 (RECIST v1.1)^18,19^ is not accurately reflecting the metastatic spread and disease progression of ILC^17^. The RECIST v1.1 guideline is used by the majority of clinical trials to evaluate tumor progression by relying on the measurability of tumoral lesions^19^. By these RECIST criteria, bone lesions are seen as non-measurable with the exception of lytic or mixed lytic-blastic bone lesions of ≥10 mm on computed tomography (CT) and magnetic resonance imaging (MRI). A number of trials are allowing the inclusion of patients with bone only disease (e.g. patients having bone metastases without evidence of any metastases in other organs) alongside patients with measurable disease according to RECIST v1.1 criteria^20–24^.

ILC is less likely to be mass-forming and has a propensity to spread to non-measurable areas such as the peritoneum^25–27^. Therefore, disease burden of ILC is likely to be underestimated by conventional imaging^5^. The greater likelihood of non-measurable disease in patients with metastatic ILC might thus lead to decreased enrollment in clinical drug trials for stage IV BC^17^. This systematic review, therefore, aims at identifying the magnitude of the gaps in ILC documentation and representation in clinical drug trials.

## Methods

### Literature search

Preferred Reporting Items for Systematic Reviews and Meta-Analyses (PRISMA) guidelines were followed to conduct the literature search and study design (Supplementary Table 1)^28^. Two reviewers (KVB and JVC) searched independently in PubMed and in the clinicaltrials.gov database to identify phase III and IV clinical trials on novel BC therapies by screening titles and abstracts. Novel drug categories were identified from reviews on novel BC treatments^29–31^. The predefined categories considered were: CDK4/6 inhibitors, ADCs, oral SERDs, PARP inhibitors, tyrosine kinase inhibitors (TKI), mTOR inhibitors, ICIs, PI3K/AKT/PTEN-inhibitors and ‘others’. Medical subject heading terms (MeSH) related to the treatments and treatment categories were used as well as names of individual drugs in the search together with the MeSH-term ‘Breast Neoplasms’ (Supplementary Table 2).

### Study selection

**Fig. 1:**
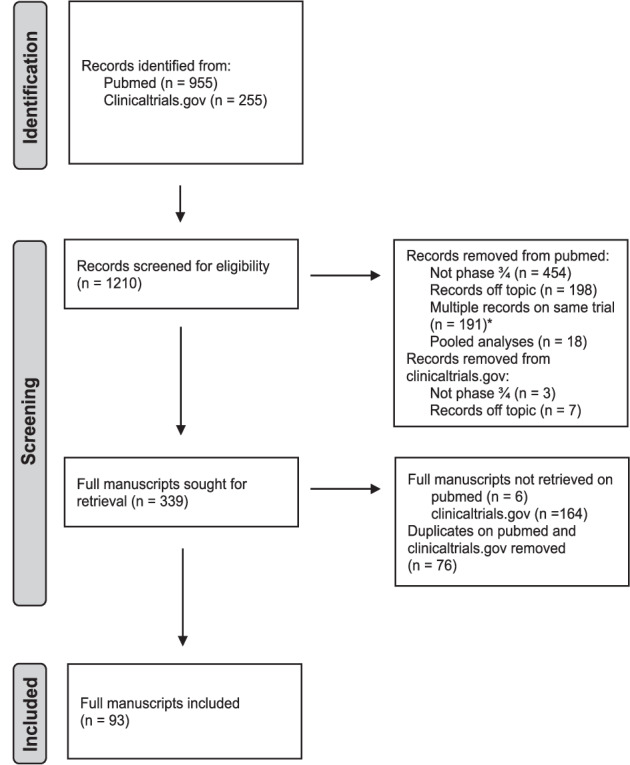
PRISMA-diagram of study selection of clinical drug trials. *An exception was made for different study arms of basket trials, 1 manuscript per study arm was allowed: 2 manuscripts of 1 basket trial (GeparQuinto, NCT00567554)^35,36^ were included in this systematic review.

Records were excluded when title, abstract or information on clinicaltrials.gov showed that it was not a phase III/IV trial. In case of multiple records on the same trial, only the record on the main publication was considered. An exception was made for basket trials investigating multiple drugs where 1 record per study arm was allowed. Pooled analyses using results of multiple trials were excluded as well as trials that were not investigating the efficacy of drugs associated with the used search term (records off topic). The PRISMA-diagram of study selection for clinical trials is shown in Fig. 1. Only trials with a full manuscript available on 31 July 2023 were considered. Therefore, records of which no available publication could be retrieved or with only an abstract or poster available at 31 July 2023, were excluded. Duplicates found on both Pubmed and clinicaltrials.gov were removed. In case there were disagreements between the reviewers, these were resolved by discussion and consensus.

### Data extraction

Inclusion and exclusion criteria reported in the manuscript and on clinicaltrials.gov were examined to see if histological subtype and measurability of disease were used as criteria. Furthermore, full manuscripts and accompanying supplementary data were analyzed to see if the number or percentage of patients with ILC was reported. In cases where the inclusion of patients with ILC was documented in the manuscript or supplementary data, the percentage of patients with ILC included was collected, the performance of central pathology for histological subtype was checked as well as the performance of specific subgroup analyses for ILC. These manuscripts were also analyzed to see if they reported on the inclusion rates of different races. Other data collected included the pharmaceutical company linked to the trial, investigated treatment arms, primary endpoints, secondary endpoints, restrictions on therapy lines and samples needed for inclusion.

### Secondary analyses

In a second stage a search was done on PubMed to see if any secondary analyses specific for patients with ILC were done for all included trials. The names and classes of the drugs as well as the names of the different trials were used together with the MeSH-term ‘Carcinoma, Lobular’ to see if any additional analyses had been done. In this stage, pooled analyses of the included trials were also searched to see if they reported on sub-analyses for patients with ILC.

Results are shown in a descriptive manner. No statistical analyses have been performed.

## Results

### Identification of the trials

In total, 93 manuscripts were included in this systematic review. The majority of the clinical trials were conducted for patients with stage IV disease (Table 1). The categories for which more than 10 manuscripts were found were CDK4/6 inhibitors, ADCs, TKIs and mTOR inhibitors. Novartis, Roche and Pfizer were each involved in ≥10 of the included trials. Supplementary Table 3 gives an overview of the acquired data per manuscript.

**Table 1:** Number of included clinical drug trials per drug category and per pharmaceutical company

	NEOADJUVANT	ADJUVANT	METASTATIC	TOTAL
DRUG CATEGORY				
-*TKI*	6	3	15	24
-*CDK4/6 INHIBITORS*	1	3	14	18
-*ADCS*	1	2	10	13
-*MTOR INHIBITORS*	1	2	10	13
-*ICI*	4	0	4	8
-*PARP INHIBITORS*	1	1	5	7
-*PI3K/AKT/PTEN-INHIBITORS*	0	0	7	7
-*OTHERS*	0	0	2	2
-*ORAL SERDS*	0	0	1	1
PHARMACEUTICAL COMPANY				
-*NOVARTIS*	1	1	24	26
-*ROCHE*	3	2	9	14
-*PFIZER*	1	2	7	10
-*GSK*	2	1	5	8
-*ASTRAZENECA*	0	1	7	8
-*LILLY*	0	1	3	4
-*MSD*	1	0	2	3
-*JIANGSU HENGRUI MEDICINE*	1	0	2	3
-*ABBVIE*	1	0	1	2
-*PUMA BIOTECHNOLOGY*	0	1	1	2
-*GILEAD SCIENCES*	0	0	2	2
-*STEMLINE THERAPEUTICS*	0	0	1	1
-*Sanofi*	0	0	1	1
-*CHIPSCREEN BIOSCIENCES*	0	0	1	1
-*MACROGENICS*	0	0	1	1
-*NO COMPANY*	4	2	4	10
TOTAL	14	11	68	93

*ADC* antibody drug conjugate, *ICI* immune checkpoint inhibitors, *SERD* selective estrogen receptor degrader, *TKI* tyrosine kinase inhibitor.

### Inclusion and exclusion criteria

ILC subtype was used as an exclusion criterion in only one trial: the NeoTRIP^32^ trial, which investigated the use of atezolizumab in the neoadjuvant setting. Measurability of disease was more often considered in the eligibility criteria. Patients with non-measurable disease were excluded in 20/93 (21.5%) trials, in 2 out of the 14 (14.3%) neoadjuvant and in 18 out of the 68 (26.5%) metastatic trials. In the metastatic setting, 19/68 (27.9%) trials additionally excluded non-measurable disease, however making an exception for patients with bone-only lesions which could be included. Therefore, restrictions based on measurability of lesions were found in 54.4% of the trials conducted in the metastatic setting.

### ILC documentation

**Fig. 2:**
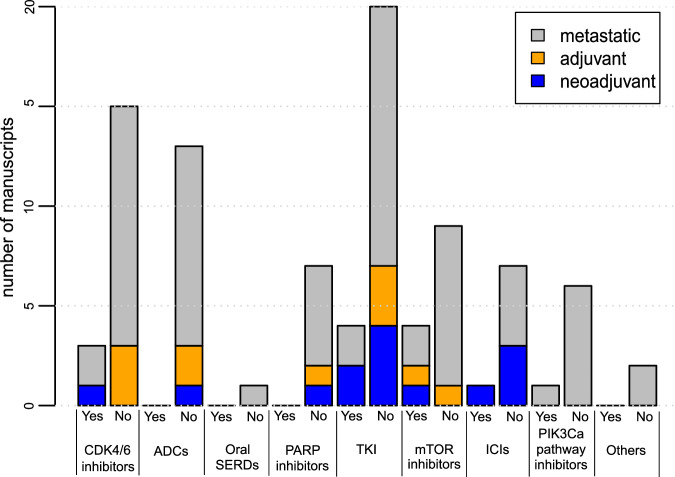
Documentation on ILC in clinical drug trials per drug category. The number of manuscripts with documentation (Yes) *vs*. without documentation (No) on ILC are demonstrated per drug category. ADC: antibody drug conjugate; ICI: immune checkpoint inhibitors; SERD: selective estrogen receptor degrader; TKI: tyrosine kinase inhibitor.

For a total of 13/93 (14.0%) of the included phase III and IV clinical drug trials, the number or percentage of included patients with ILC could be found in the manuscript or accompanying supplementary data. In the neoadjuvant setting, 5/14 (35.7%) trials documented the inclusion of ILC. For adjuvant and metastatic trials, documentation on ILC was only found in 1/11 (9.1%) and 7/68 (10.3%) of the trials, respectively. Patients with luminal hormone receptor-positive (HR+) HER2 negative BC were of interest in 49/93 (52.7%) manuscripts (Supplementary Table 3). In 10/49 (20.4%) of these manuscripts there was documentation on ILC inclusion. In Fig. 2, the documentation on ILC per drug category is shown for neoadjuvant, adjuvant and metastatic drug trials. Several drug categories had no documentation on ILC in any of the published manuscripts. These categories included oral SERDs, ADCs and PARP inhibitors.

**Fig. 3:**
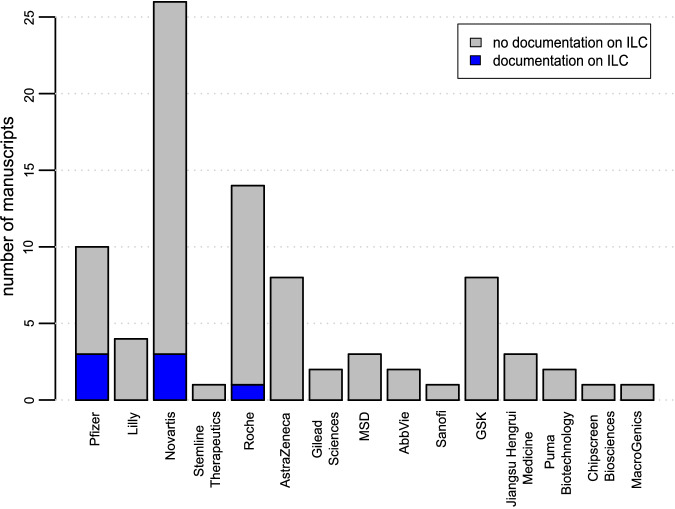
Documentation on ILC in clinical drug trials per pharmaceutical company. Number of manuscripts per pharmaceutical company involved in trial conduct. No company was involved in 10 trials and multiple companies were involved in 3 trials.

In 83/93 (89.2%) trials, at least one pharmaceutical company was involved. In 3 trials, 2 companies were involved. Figure 3 gives an overview on how many trials per pharmaceutical company reported on ILC inclusion in their original publications. For the majority of companies, no manuscript reported on ILC. Pfizer had a reporting rate of 30% (3/10 trials), Novartis of 11.5% (3/26 trials) and Roche of 7.1% (1/14 trials).

### ILC central pathology, representation, and sub-analyses

The 13 manuscripts with documentation on ILC are summarized in Table 2. The performance of central pathology to review the histological subtype, was not reported in any of these manuscripts. Non-measurable disease was an exclusion criterion in two of the trials^33,34^ and non-measurable disease with exception of bone-only disease was excluded in two other trials^23,24^. The inclusion rate of patients with ILC varied between 2.0 and 26.0%. In the case of a population with HR + HER2- BC, the inclusion rate varied between 3.8 and 26.0%. Specific sub-analyses for patients with ILC were only performed in 3/13 (23.1%) trials^35–37^. Only 6/13 (46.2%) had reporting on the race of included patients^23,24,33,34,37,38^. However, 3 of these manuscripts only differentiated between Asian race and ‘others’^24,34,37^.

**Table 2:** Clinical drug trial with documentation on ILC available

SETTING	TRIAL	DRUG	PATIENT POPULATION	% ILC INCLUDED	SUB-ANALYSIS	CENTRAL PATHOLOGY
Neoadjuvant	SAFIA^59^	palbociclib (CDK4/6i)	HR+HER2−	12.0	No	No
	IMpassion031^33^^,a^	atezolizumab (ICI)	TNBC	2.0	No	No
	GeparQuinto Lapatinib^36^	lapatinib (TKI)	HR− HER2+ or HR+HER2+ if cN+	2.8	Yes	No
	EPHOS B^60^	lapatinib (TKI)	HER2+	4.0	No	No
	GeparQuinto Everolimus^35^	everolimus (mTORi)	HR− HER2+ or HR+HER2+ if cN+	10.8	Yes	No
Adjuvant	MAINtenance Afinitor^61^	everolimus (mTORi)	HR+HER2−	16.3	No	No
Metastatic	PALOMA 2^23,b]^	palbociclib (CDK4/6i)	HR+HER2−	14.7	No	No
	PALOMA 4^24,b]^	palbociclib (CDK4/6i)	HR+HER2−	3.8	No	No
	NCT00281658^34,a]^	lapatinib (TKI)	HER2+	4.7	No	No
	DETECT III^62^	lapatinib (TKI)	HER2− with HER2+ CTCs	9.8	No	No
	BELLE-2^38^	buparlisib (PI3Ki)	HR+HER2−	13.0	No	No
	INPRES^63^	everolimus (mTORi)	HR+HER2−	26.0	Yes	No
	IMPROVE^37^	everolimus (mTORi)	HR+HER2−	24.7	No	No

*cN* clinical nodal status, *CTCs* circulating tumor cells, *HR* hormone receptor, *TNBC* triple negative breast cancer.

^a^Exclusion of non-measurable disease.

^b^Exclusion of non-measurable disease with the exception of bone-only disease.

### Secondary analyses

Secondary analyses specific for patients with ILC were not found for any of the included trials. The search on the included drugs names and classes neither uncovered any secondary analyses for ILC that were using trial data. Pooled analyses with comparison of efficacy for ILC vs. IBC-NST were only found for CDK4/6 inhibitors^39,40^.

## Discussion

This systematic review demonstrates that ILC is largely disregarded in many clinical trials since only 13 out of 93 manuscripts (14.0%) reported how many patients with ILC were included. Trials including patients with luminal breast cancer did a little better with 20.4% of the manuscripts reporting on ILC inclusion. Specific sub-analyses for ILC on prospective data to evaluate the efficacy of novel BC treatments are seldom performed. Future reporting of the proportion of patients with ILC included will not guarantee the possibility of performing sub-analyses in specific clinical trials, since the subset might be too small. However, it is still valuable to collect data on histological subtypes and report the proportion of ILC since it will indicate if patients with ILC were underrepresented or well represented in the clinical trial. Furthermore, pooled analyses on different histological subtypes can only be performed if the data is collected and made available.

Even when ILC is reported, no details are given on the specific ILC subtypes that the included patients were diagnosed with. Classic ILC only represents ~55% of all ILC cases and worse outcomes have been described for patients with non-classic types ILC compared to classic ILC^1,41^. At present, no clear histological criteria have been defined to recognize and diagnose the different ILC subtypes^7^.

This review shows that these prospective trials do not perform central pathology to confirm histological subtypes. However, previous studies have revealed that 34-40% of the tumors diagnosed as ILC locally are not confirmed by central pathology review, while ~2% of the tumors that are locally subtyped as non-ILC are centrally reclassified as ILC^8,9^. This could be explained by the great variability in the definition of pathological diagnosis of ILC^1^, which was recently documented in a worldwide survey^42^. Efforts to harmonize and facilitate the pathological diagnosis of ILC are currently being undertaken by the pathology working group of the European Lobular Breast Cancer Consortium (www.elbcc.org).

Since patients with ILC are rarely considered in phase III/IV clinical drug trials, patients with ILC and their treating physicians are mainly relying on results in the global BC population and limited retrospective analyses in ILC populations to estimate the clinical benefit. Concerning CDK4/6 inhibitors, Gao et al. reported in their pooled analyses that CDK4/6 inhibitors were as effective for patients with ILC as for patients with IBC-NST in the metastatic setting^39,40^. Unfortunately, histological subtyping was missing for more than half of the patients in these pooled analyses, and as a result, the analyses for ILC were performed only on a small number of patients (*n* = 269). Several retrospective studies came to a similar conclusion that the benefit of CDK4/6 inhibitors is similar for metastatic ILC in comparison to IBC-NST^43,44^.

Limited data exist that there is no significant difference in the benefit of everolimus between patients with metastatic ILC vs. IBC-NST^43^. A retrospective study concluded that patients with ILC benefit more from the combination of exemestane with everolimus than the combination of palbociclib with fulvestrant when used in second line of metastatic treatment in patients with hormone-resistant disease^45^. However, no comparison with patients with IBC-NST was made and inclusion was limited to 48 patients receiving palbociclib with fulvestrant and 26 receiving everolimus with exemestane. In one study, no difference in progression-free survival for alpelisib between patients with ILC and IBC-NST was observed, however, only 9 patients with ILC were included in this analysis^43^. To our knowledge, no additional retrospective analyses evaluating other novel BC treatments for patients with ILC are available. Several drugs included in this systematic review are still under investigation and not commercially available. It is highly regrettable that patients with ILC need to wait until real-world data with enough follow-up are available to evaluate the benefit of the different novel BC treatments.

Some prospective trials that were either specifically designed for patients with ILC or have a clear aim to include patients with ILC for retrospective sub-analyses, are currently ongoing^5^. The majority of these trials are phase II and were thus not included in this systematic review. ROS1-inhibitors which exhibit synthetic lethality with *CDH1* mutations are currently under investigation in the neoadjuvant and metastatic setting^46,47^. Other trials in the metastatic setting target BC with *HER2* and *HER3* mutations which are more common in ILC compared to IBC-NST^2,48,49^. In the phase 2 MutHER trial, 42.5% of the included patients were diagnosed with ILC^49^. A higher clinical benefit rate (61.5% vs 18.2%) of neratinib was described for patients with ILC in comparison to patients with IBC-NST. The use of atezolizumab for patients with ILC was investigated in the GELATO trial^50^, which was terminated prematurely due to short-lived treatment responses. The responses they observed were mostly in patients with triple-negative ILC. Clearly, there is a need for phase 3 and 4 trials dedicated to study drug efficiency in patients with ILC.

Concerning the manuscripts reporting on ILC, the percentage of ILC patients varied between 2.0% and 26.0%. However, if we consider the clinical subtype (HR+, triple negative or HER2+) that was targeted in the trials, the representation of ILC in these trials was quite similar to what is seen in clinical practice. Triple negative and HER2 positive tumors are found in 2.0–9.0% and 3.0–13.0% of ILC, respectively^1^. A noteworthy exception was however seen in PALOMA 4, which included only 3.8% patients with ILC although this trial focused on HR+ HER2− tumors^24^. This study was conducted in an Asian population and ILC has been reported to be less often diagnosed in Asia as compared to Europe^51^.

Although representation in those 13 manuscripts seemed to reflect the relative incidence of ILC compared to IBC-NST in clinical practice, only 4 of these trials had inclusion restrictions based on measurability of lesions. In total, 39/93(41.9%) manuscripts had inclusion and exclusion criteria involving measurability. Since documentation on ILC was so poor in these trials, no conclusion can be made if the presence of inclusion or exclusion criteria on measurability affects the inclusion rate of patients with ILC. Conventional imaging has its limitations in quantifying the disease burden in the case of metastatic ILC. [^18^F]2-fluoro-2-deoxy-D-glucose (^18^F-FDG)–positron emission tomography (PET)/CT sensitivity is limited by the decreased uptake of glucose by the metabolically less active ILC lesions^52^. Although whole-body diffusion-weighted magnetic resonance imaging (WB-DWI/MRI) has superiority over other imaging techniques to detect peritoneal metastases, this infiltration is often diffuse and not measurable^53^. Additionally, WB-DWI/MRI is not widely available and routinely used in clinical trials. Current RECIST criteria put the emphasis on measurable lesions of at least 10 mm for non-nodal and 15 mm for nodal lesions^18^. This does not reflect the unique metastatic pattern and diffuse infiltration of ILC^25,26^. Artificial intelligence might help in quantifying the total disease burden in patients with BC and therefore evaluating progression, even in cases of non-measurable disease^54^.

This systematic review has several limitations. Only clinical trials registered in Pubmed or clinicaltrials.gov were included. Data on clinicaltrials.gov depend for a large part on updates from the clinical trial groups involved and could therefore have been incomplete at the time of our search. Only the main publication was included per trial. Information on patients with ILC might have been available in other reports on these trials. We therefore also performed a secondary search to identify secondary analyses done for patients with ILC.

As shown in this systematic review, patients with ILC are often neglected by clinical investigators and pharmaceutical industries. This leads to a significant unmet clinical need, as the global age-standardized incidence (ASI) of BC, according to GLOBOCAN 2020 was 47.8 per 100.000 women^55^ and estimating that ILC represents approximately 15% of all BC^16^, the incidence of ILC would be around 7.2 per 100.000 women. This is comparable to the number of women affected by stomach cancer (ASI 7.0 per 100.000) and even higher as compared to the incidence of ovarian (ASI 6.6 per 100.000) and liver cancers (ASI 5.2 per 100.000) in women^55^. Other rarer histological subtypes are most probably even less documented in clinical trials.

The problem of unsatisfactory inclusion of other important patient subgroups is reflected by the underrepresentation of e.g., racial minorities, elderly patients, and male patients^56–58^. For patients belonging to multiple minority subgroups, the treatment benefit of the different drugs is even more unclear. Considering the manuscripts with documentation on ILC, less than half of them (46.2%) reported inclusion rates per race^24,34,37^. As a result, women diagnosed with ILC belonging to a racial minority group are highly unsure of the treatment benefit in case novel drugs are implemented in their treatment.

It is important to acknowledge the poor documentation and underrepresentation of ILC in clinical trials since it impedes the personalized treatment of all patients diagnosed with ILC. Clinical investigators and pharmaceutical industries should increase their efforts to include patients with ILC in trials. Inclusion and exclusion based on RECIST criteria need to be reevaluated for that purpose. Furthermore, all clinical trials should aim to do prospective analyses dedicated to ILC, so that these patients do not have to rely solely on limited retrospective analyses. In peer-reviewed journals, one could insist on reporting of the percentage of patients with ILC involved in these trials. It is clear that urgent efforts are needed to accommodate patients with ILC.

To conclude, patients with ILC are often overlooked in clinical drug trials. Documentation on the number of patients with ILC included is poor. The few trials reporting on ILC inclusion lack specific sub-analyses on ILC and do not report on central pathology to confirm histological subtype. Eligibility criteria and definitions of treatment response need to be re-evaluated to better reflect the unique biology of ILC. It is critical that patients with ILC are considered by clinical investigators and pharmaceutical industries.

### Reporting summary

Further information on research design is available in the Nature Research Reporting Summary linked to this article.

### Supplementary information

Appendix: Reporting on invasive lobular breast cancer in clinical drug trials – a systematic review

Reporting Summary

## Data Availability

All data has been made available in the manuscript and supplementary material.

## Code availability

Given the descriptive nature of the manuscript, no code was used.
