# Supplementary material for: Reporting on invasive lobular breast cancer in clinical trials: a systematic review
Source: NPJ Breast Cancer. 2024 Mar 20;10:23. doi: 10.1038/s41523-024-00627-5 (PMC10954721; doi:10.1038/s41523-024-00627-5)
Supplement: Supplementary file 1 — Appendix: Reporting on invasive lobular breast cancer in clinical drug trials – a systematic review [file 41523_2024_627_MOESM1_ESM.pdf]

Appendix: Reporting on invasive lobular breast cancer in clinical drug trials – a systematic review

Van Baelen K., Van Cauwenberge J. *et al.*

Overview:

- Supplementary Table 1: PRISMA checklist
- Supplementary Table 2: Search terms clinical drug trials
- Supplementary Table 3: Acquired data per included clinical drug trial

Supplementary Table 1: PRISMA checklist

Section and Topic	Item #	Checklist item	Location where item is reported
**TITLE**			
Title	1	Identify the report as a systematic review.	Title
**ABSTRACT**			
Abstract	2	See the PRISMA 2020 for Abstracts checklist.	Abstract
**INTRODUCTION**			
Rationale	3	Describe the rationale for the review in the context of existing knowledge.	Introduction
Objectives	4	Provide an explicit statement of the objective(s) or question(s) the review addresses.	Introduction
**METHODS**			
Eligibility criteria	5	Specify the inclusion and exclusion criteria for the review and how studies were grouped for the syntheses.	Methods
Information sources	6	Specify all databases, registers, websites, organisations, reference lists and other sources searched or consulted to identify studies. Specify the date when each source was last searched or consulted.	Methods
Search strategy	7	Present the full search strategies for all databases, registers and websites, including any filters and limits used.	Methods and supplementary data
Selection process	8	Specify the methods used to decide whether a study met the inclusion criteria of the review, including how many reviewers screened each record and each report retrieved, whether they worked independently, and if applicable, details of automation tools used in the process.	Methods
Data collection process	9	Specify the methods used to collect data from reports, including how many reviewers collected data from each report, whether they worked independently, any processes for obtaining or confirming data from study investigators, and if applicable, details of automation tools used in the process.	Methods
Data items	10a	List and define all outcomes for which data were sought. Specify whether all results that were compatible with each outcome domain in each study were sought (e.g. for all measures, time points, analyses), and if not, the methods used to decide which results to collect.	Methods and supplementary data
	10b	List and define all other variables for which data were sought (e.g. participant and intervention characteristics, funding sources). Describe any assumptions made about any missing or unclear information.	Methods and supplementary data
Study risk of bias assessment	11	Specify the methods used to assess risk of bias in the included studies, including details of the tool(s) used, how many reviewers assessed each study and whether they worked independently, and if applicable, details of automation tools used in the process.	NA
Effect measures	12	Specify for each outcome the effect measure(s) (e.g. risk ratio, mean difference) used in the synthesis or presentation of results.	NA
Synthesis methods	13a	Describe the processes used to decide which studies were eligible for each synthesis (e.g. tabulating the study intervention characteristics and comparing against the planned groups for each synthesis (item #5)).	Methods and supplementary data
	13b	Describe any methods required to prepare the data for presentation or synthesis, such as handling of missing summary statistics, or data conversions.	NA
	13c	Describe any methods used to tabulate or visually display results of individual studies and syntheses.	NA
	13d	Describe any methods used to synthesize results and provide a rationale for the choice(s). If meta-analysis was performed, describe the model(s), method(s) to identify the presence and extent of statistical heterogeneity, and software package(s) used.	NA
	13e	Describe any methods used to explore possible causes of heterogeneity among study results (e.g. subgroup analysis, meta-regression).	NA

Section and Topic	Item #	Checklist item	Location where item is reported
	13f	Describe any sensitivity analyses conducted to assess robustness of the synthesized results.	NA
Reporting bias assessment	14	Describe any methods used to assess risk of bias due to missing results in a synthesis (arising from reporting biases).	NA
Certainty assessment	15	Describe any methods used to assess certainty (or confidence) in the body of evidence for an outcome.	NA
**RESULTS**			
Study selection	16a	Describe the results of the search and selection process, from the number of records identified in the search to the number of studies included in the review, ideally using a flow diagram.	Figure 1 and results
	16b	Cite studies that might appear to meet the inclusion criteria, but which were excluded, and explain why they were excluded.	NA
Study characteristics	17	Cite each included study and present its characteristics.	Supplementary data
Risk of bias in studies	18	Present assessments of risk of bias for each included study.	NA
Results of individual studies	19	For all outcomes, present, for each study: (a) summary statistics for each group (where appropriate) and (b) an effect estimate and its precision (e.g. confidence/credible interval), ideally using structured tables or plots.	NA
Results of syntheses	20a	For each synthesis, briefly summarise the characteristics and risk of bias among contributing studies.	NA
	20b	Present results of all statistical syntheses conducted. If meta-analysis was done, present for each the summary estimate and its precision (e.g. confidence/credible interval) and measures of statistical heterogeneity. If comparing groups, describe the direction of the effect.	NA
	20c	Present results of all investigations of possible causes of heterogeneity among study results.	NA
	20d	Present results of all sensitivity analyses conducted to assess the robustness of the synthesized results.	NA
Reporting biases	21	Present assessments of risk of bias due to missing results (arising from reporting biases) for each synthesis assessed.	NA
Certainty of evidence	22	Present assessments of certainty (or confidence) in the body of evidence for each outcome assessed.	NA
**DISCUSSION**			
Discussion	23a	Provide a general interpretation of the results in the context of other evidence.	Discussion
	23b	Discuss any limitations of the evidence included in the review.	Discussion
	23c	Discuss any limitations of the review processes used.	Discussion
	23d	Discuss implications of the results for practice, policy, and future research.	Discussion
**OTHER INFORMATION**			
Registration and protocol	24a	Provide registration information for the review, including register name and registration number, or state that the review was not registered.	Not registered
	24b	Indicate where the review protocol can be accessed, or state that a protocol was not prepared.	Not prepared
	24c	Describe and explain any amendments to information provided at registration or in the protocol.	NA
Support	25	Describe sources of financial or non-financial support for the review, and the role of the funders or sponsors in the review.	Funding
Competing interests	26	Declare any competing interests of review authors.	Conflict of interest
Availability of data, code and	27	Report which of the following are publicly available and where they can be found: template data collection forms; data extracted from included studies; data used for all analyses; analytic code; any other materials used in the review.	Supplementary data

Section and Topic	Item #	Checklist item	Location where item is reported
other materials			

From: Page MJ, McKenzie JE, Bossuyt PM, Boutron I, Hoffmann TC, Mulrow CD, et al. The PRISMA 2020 statement: an updated guideline for reporting systematic reviews. BMJ 2021;372:n71. doi: 10.1136/bmj.n71

For more information, visit: http://www.prisma-statement.org/

Supplementary Table 2: Search terms clinical drug trials

Search terms	Clinicaltrials.gov	Pubmed
**General search terms used**		
	i. Condition or disease: Breast Cancer<br>ii. Study Phase: Phase 3, Phase 4	"Breast Neoplasms"[Mesh] AND article type: 'Clinical Trial, phase III' OR 'Clinical Trial, phase IV'
**Additional search terms used per drug class**		
CDK4/6 inhibitors	Other terms: "CDK4/6 inhibitor" OR "palbociclib" OR "abemaciclib" OR "ribociclib"	"palbociclib" [Supplementary Concept] OR "ribociclib" [Supplementary Concept] OR "abemaciclib" [Supplementary Concept]
Oral SERDs	Other terms: "SERD" OR "selective estrogen receptor degrader" OR "amcenestrant" OR "elacestrant" OR "imlunestrant" OR "camizestrant" OR "giredestrant"	"selective estrogen receptor degrader" [Supplementary Concept] OR "oral selective estrogen receptor degrader" [Supplementary Concept] OR "elacestrant" [Supplementary Concept] OR "amcenestrant" [Supplementary Concept] OR "imlunestrant" [Supplementary Concept] OR "camizestrant" [Supplementary Concept] OR "giredestrant" [Supplementary Concept] OR "AZD9833" [Supplementary Concept]
Antibody drug conjugates	Other terms: "Antibody-drug conjugates" OR "T-DM1" OR "trastuzumab emtansine" OR "T-DXd" OR "trastuzumab deruxtecan" OR "sacituzumab govitecan"	"immunoconjugates" [Mesh] OR "sacituzumab govitecan" [Supplementary Concept] OR "trastuzumab deruxtecan" [Supplementary Concept] OR "Ado-trastuzumab Emtansine" [Mesh]
Immune checkpoint inhibitors	Other terms: "Immune Checkpoint inhibitor" OR "pembroluzimab" OR "PDL-1 inhibitor" OR atezolizumab" OR "nivolumab" OR "ipilimumab"	"Immune Checkpoint Inhibitors"[Mesh] OR "pembrolizumab" [Supplementary Concept] OR "atezolizumab" [Supplementary Concept] OR "nivolumab"[Mesh] OR "ipilimumab"[Mesh]
PARP inhibitors	Other terms: "PARP inhibitors" OR "olaparib" OR "niraparib" OR "talazoparib"	"Poly(ADP-ribose) Polymerase Inhibitors"[Mesh] OR "olaparib" [Supplementary Concept] OR "talazoparib" [Supplementary Concept] OR "niraparib" [Supplementary Concept] OR "veliparib" [Supplementary Concept] OR "iniparib" [Supplementary Concept]
Tyrosine kinase inhibitors	Other terms: "Tyrosine Kinase Inhibitors" OR "lapatinib" OR "neratinib" OR "pyrotinib"	"Tyrosine Kinase Inhibitors"[Mesh] OR "lapatinib"[Mesh] OR "neratinib" [Supplementary Concept] OR "pyrotinib" [Supplementary Concept]
mTOR inhibitors	Other terms: "mTOR inhibitors" OR "everolimus" OR "temsirolimus" OR "sirolimus"	"everolimus"[Mesh] OR "temsirolimus" [Supplementary Concept] OR "sirolimus" [Supplementary Concept]
PI3K pathway inhibitors	Other terms: "PIK3CA inhibitors" OR "alpelisib" OR "buparlisib" OR "ipatasertib" OR "taselisib" OR "capivasertib"	"alpelisib" [Supplementary Concept] OR "NVP-BKM120" [Supplementary Concept] OR "ipatasertib" [Supplementary Concept] OR "2-(3-(2-(1-isopropyl-3-methyl-1H-1,2-4-triazol-5-yl)-5,6-

		dihydrobenzo(f)imidazo(1,2-d)(1,4)oxazepin-9-yl)-1H-pyrazol-1-yl)-2-methylpropanamide" [Supplementary Concept] OR "capivasertib"
Others	Other terms: "margetuximab" OR "tucidinostat"	"margetuximab" [Supplementary Concept] OR "N-(2-amino-5-fluorobenzyl)-4-(N-(pyridine-3-acrylyl)aminomethyl)benzamide" [Supplementary Concept]

SERD: selective estrogen receptor degrader

Supplementary Table 3: Acquired data per included clinical drug trial

Setting	Drug	Trial	Source	Company	Enrollment	Treatment arms	Primary endpoint	Secondary endpoints	Exclusion based on non-measurable disease	Exclusion of ILC	Central pathology for ILC	Is number or % ILC reported	%ILC	Subgroup analyses on ILC performed
**CDK4/6 inhibitors**														
Neoadjuvant	Palbociclib	SAFIA[1]	Clinicaltrials.gov<br>Pubmed	Pfizer	354	°fulvestrant +/- goserelin + palbociclib<br>°fulvestrant +/- goserelin + placebo	pCR	°Radiological response<br>°Rate of BCS<br>°Safety/ Tolerability<br>°DFS<br>°OS	No	No	No	Yes	12.0	No
Adjuvant	Abemaciclib	MONARCHE[2]	Clinicaltrials.gov<br>Pubmed	Lilly	5637	°endocrine treatment (TPC) + abemaciclib<br>°endocrine treatment (TPC)	iDFS	°iDFS in case of Ki67 ≥20%<br>°DRFS<br>°OS<br>°PK<br>°FACT-B, FACIT-F, EQ-5D-5L	NA	No	No	No	NA	NA
	Palbociclib	PENELOPE-B[3]	Clinicaltrials.gov<br>Pubmed	Pfizer	1250	°endocrine treatment (TPC) + palbociclib<br>°endocrine treatment (TPC) + placebo	iDFS	°iDFS excluding second non-breast cancers<br>°DDFS<br>°OS<br>°iDFS in luminal B<br>°Compliance/ Safety<br>°Patient reported outcomes, QALY<br>°AUC<br>°Correlation between	NA	No	No	No	NA	NA

								exposure/efficacy and/or safety						
	Palbociclib	PALLAS[4]	Clinicaltrials.gov<br>Pubmed	Pfizer	5796	°endocrine treatment (TPC) + palbociclib<br>°endocrine treatment (TPC)	iDFS	°iDFS excluding second non-breast cancers<br>°DRFS<br>°OS<br>°LRRFS	NA	No	No	No	NA	NA
Metastatic	Abemaciclib	MONARCH2[5]	Clinicaltrials.gov<br>Pubmed	Lilly	669	°fulvestrant + abemaciclib<br>°fulvestrant + placebo	PFS	°OS<br>°ORR<br>°DOR<br>°DCR<br>°CBR<br>°mBPI-sf<br>°PK<br>°EQ-5D-5L, EORTC QLQ-C30, EORTC QLQ-BR23	Yes (exception of bone only disease)	No	No	No	NA	NA
	Abemaciclib	MONARCH3[6]	Clinicaltrials.gov<br>Pubmed	Lilly	493	°AI + abemaciclib<br>°AI + placebo	PFS	°OS<br>°ORR<br>°DOR<br>°DCR<br>°CBR<br>°ES-5D-5L, EORTC QLQ-C30; EORTC QLQ-BR23<br>°PK	Yes (exception of bone only disease)	No	No	No	NA	NA
	Abemaciclib	MONARCH Plus[7]	Clinicaltrials.gov<br>Pubmed	Lilly	463	°AI + abemaciclib<br>°AI + placebo<br>°fulvestrant + abemaciclib<br>°fulvestrant + placebo	PFS	°OS<br>°ORR<br>°DOR<br>°DCR<br>°CBR<br>°EORTC QLQ-C30<br>°PK	Yes (exception of bone only disease)	No	No	No	NA	NA
	Palbociclib	PALOMA2[8]	Clinicaltrials.gov<br>Pubmed	Pfizer	666	°letrozole + palbociclib<br>°letrozole + placebo	PFS	°ORR<br>°DOR<br>°CBR<br>°PFS by biomarkers<br>°QTc<br>°Ctrough<br>°EQ-5D, FACT-B<br>°TEAE<br>°OS<br>°Survival probability	Yes (exception of bone only disease)	No	No	Yes	14.7	No

								°CTCAE						
	Palbociclib	PALOMA3[9]	Clinicaltrials.gov<br>Pubmed	Pfizer	521	°fulvestrant + palbociclib<br>°fulvestrant + placebo	PFS	°OS<br>°ORR<br>°DOR<br>°CBR<br>°Survival probabilities<br>°Ctrough<br>°EORTC QLQ-C30, EORTC QLQ-BR23, EQ-5D<br>°TTD<br>°TEAE	Yes (exception of bone only disease)	No	No	No	NA	NA
	Palbociclib	PALOMA4[10]	Clinicaltrials.gov<br>Pubmed	Pfizer	340	°letrozole + palbociclib<br>°letrozole + placebo	PFS	°ORR<br>°DOR<br>°CBR<br>°OS<br>°Survival Probability<br>°TEAE<br>°CTCAE<br>°Ctrough<br>°EQ-5D, FACT-B<br>°% positive cells for Ki67<br>°Detection in ER	Yes (exception of bone only disease)	No	No	Yes	3.8	No
	Palbociclib	PADA-1[11]	Clinicaltrials.gov<br>Pubmed	Pfizer	1017	°AI + palbociclib<br>°fulvestrant + palbociclib	°TEAE<br>°PFS	°Time to strategy failure<br>°Chemotherapy-free survival<br>°EORTC QLQ-C30<br>°Other line of therapy<br>°OS	Yes (exception of bone only disease)	No	No	No	NA	NA
	Palbociclib	PEARL[12]	Clinicaltrials.gov<br>Pubmed	Pfizer	693	°fulvestrant/exemestane + palbociclib<br>°capecitabine	PFS	°ORR<br>°CBR<br>°DOR<br>°OS<br>°TEAE	Yes (exception of bone only disease)	No	No	No	NA	NA
	Ribociclib	MONALEESA2[13]	Clinicaltrials.gov<br>Pubmed	Novartis	668	°letrozole + ribociclib<br>°letrozole + placebo	PFS	°ORR<br>°OS<br>°CBR<br>°Time to deterioration ECOG	Yes (exception of bone only disease)	No	No	No	NA	NA

								performance<br>status<br>°Safety and<br>Tolerability<br>°EORTC QLQ-C30<br>°QTc						
	Ribociclib	MONALEESA3[14]	Clinicaltrials.gov<br>Pubmed	Novartis	726	°fulvestrant + ribociclib<br>°fulvestrant + placebo	PFS	°OS<br>°ORR<br>°TTD<br>°ECOG<br>performance<br>status<br>°Safety and<br>Tolerability<br>°EORTC QLQ-C30<br>°CBR<br>°TTR<br>°DOR	Yes<br>(exception<br>of bone<br>only<br>disease)	No	No	No	NA	NA
	Ribociclib	MONALEESA7[15]	Clinicaltrials.gov<br>Pubmed	Novartis	672	°AI/tamoxifen + goserelin + ribociclib<br>°AI/tamoxifen + goserelin + placebo	PFS	°OS<br>°ORR<br>°CBR<br>°Safety and<br>Tolerability<br>°TTR<br>°DOR<br>°TTD<br>°ECOG<br>performance<br>status<br>°EORTC QLQ-C30	Yes<br>(exception<br>of bone<br>only<br>disease)	No	No	No	NA	NA
	Ribociclib	RIBECCA[16]	Clinicaltrials.gov<br>Pubmed	Novartis	502	°letrozole + ribociclib in postmenopausal women or in men<br>°letrozole + goserelin + ribociclib in pre/perimenopausal women<br>°letrozole +/- goserelin + ribociclib in pretreated women or men	CBR	°PFS<br>°OS<br>°ORR<br>°EORTC QLQ-C30,<br>EORTC QLQ-BR23<br>°TTD<br>°EORTC Global<br>Health Status<br>°TEAE	No	No	No	No	NA	NA
	Ribociclib	COMPLEEMENT1[17]	Clinicaltrials.gov<br>Pubmed	Novartis	3246	°letrozole +/- goserelin/leuprolide + ribociclib	°TEAE<br>°SEA	°TTP<br>°ORR<br>°CBR<br>°FACT-B	No	No	No	No	NA	NA

	Ribociclib	BioItaLEE[18]	Clinicaltrials.gov<br>Pubmed	Novartis	287	°ribociclib + letrozole<br>°alpelisib + fulvestrant	% of patients with ctDNA alterations	°Change in TK1 concentrations<br>°Change in TMB<br>°% of patients with mutations<br>°% of patients with alterations in liquid biopsy vs. tissue biopsy<br>°Micro-environment parameters<br>°TTP<br>°adverse events<br>°ORR<br>°CBR	No	No	No	No	NA	NA
**Oral SERDs**														
Metastatic	Elacestrant	EMERALD III[19]	Clinicaltrials.gov<br>Pubmed	Stemline therapeutic s	477	°SOC (AI /fulvestrant)<br>°elacestrant	PFS	°OS<br>°BICR-assessed PFS and OS<br>°PFS assessed by the investigator<br>°ORR<br>°DOR<br>°CBR<br>°Safety and Tolerability	Yes (exception of bone only disease)	No	No	No	NA	NA
**Antibody drug conjugates**														
Neoadjuvant	T-DM1	KRISTINE[20]	Clinicaltrials.gov<br>Pubmed	Roche	444	°trastuzumab + pertuzumab<br>°T-DM1 + pertuzumab	pCR	°OS<br>°Rate of BCS<br>°EFS<br>°iDFS<br>°EORTC QLQ-C30, EORTC QLQ-BR23 modified<br>°Cmax<br>°Cmin<br>°Plasma DM1 concentrations, Serum DM1 catabolites concentrations<br>°TEAE<br>°ATA	No	No	No	No	NA	NA

Adjuvant	T-DM1	KAITLIN[21]	Clinicaltrials.gov<br>Pubmed	Roche	1846	°AC followed by trastuzumab + pertuzumab + taxane<br>°AC followed by T-DM1 + pertuzumab	iDFS	°IDFS + Second Primary Non-Breast Cancer<br>°DFS<br>°DRFI<br>°OS<br>°TEAE<br>°% with decrease LVEF<br>°EORTC QLQ-C30, EORTC QLQ-BR23	NA	No	No	No	NA	NA
	T-DM1	KATHERINE[22]	Clinicaltrials.gov<br>Pubmed	Roche	1487	°trastuzumab<br>°T-DM1	iDFS	°IDFS + Second Primary Non-Breast Cancer<br>°DFS<br>°OS<br>°DRFI<br>°TEAE<br>°Cardiac dysfunction<br>°EORTC QLQ-C30, EORTC QLQ-BR23<br>°Serum concentrations, plasma concentrations of DM1<br>°T-DM1 Exposure<br>°ATA	NA	No	No	No	NA	NA
Metastatic	T-DM1	EMILIA[23]	ClinicalTrials.gov<br>Pubmed	Roche	991	°T-DM1<br>°lapatinib + capecitabine	°% with PD by IRC<br>°PFS by IRC<br>°% death<br>°OS<br>°% alive at year 1, % alive at year 2	°% with PD by Investigator<br>°PFS by Investigator<br>°ORR<br>°DOR<br>°CBR<br>°% Treatment Failure<br>°% Symptom progression<br>°Time to Symptom progression	No	No	No	No	NA	NA
	T-DM1	TH3RESA[24]	ClinicalTrials.gov<br>Pubmed	Roche	602	°T-DM1<br>°TPC	°PFS<br>°OS	°ORR<br>°DOR	No	No	No	No	NA	NA

								°6m and 1y Survival<br>°EORTC QLQ-BM22						
	T-DM1	KAMILLA[25]	ClinicalTrials.gov<br>Pubmed	Roche	2185	°T-DM1 all participants<br>°T-DM1 Asian participants	AEPI	°AESI<br>°PFS<br>°OS<br>°ORR<br>°CBR<br>°DOR<br>°TTR<br>°Number and Type of Hospitals Visits	No	No	No	No	NA	NA
	T-DM1	MARIANNE[26]	ClinicalTrials.gov<br>Pubmed	Roche	1095	°trastuzumab + taxane<br>°T-DM1 + pertuzumab<br>°T-DM1 + pertuzumab placebo	°% Death and PD by IRC<br>°PFS by IRC	°% Death prior to clinical cut off<br>°OS<br>°% Death and PD by Investigator<br>°PFS by Investigator<br>°% with Treatment Failure<br>°TTF<br>°1y Survival<br>°Grade 3 TEAE<br>°% Death at 2y<br>°OS 2y<br>°CTCAE<br>°ECOG Performance status<br>°Hospitalization days<br>°% Hospitalizations<br>°ORR<br>°CBR<br>°FACT-TaxS<br>°FACT-C, HRQOL, FACT-B, RSCL, WPAI<br>°Outcomes in Low HER2 mRNA levels	No	No	No	No	NA	NA
	T-DM1	ELAINA[27]	Clinicaltrials.gov	Roche	200	°T-DM1<br>°lapatinib + capecitabine	PFS	°ORR<br>°DOR	No	No	No	No	NA	NA

								°OS<br>°Adverse events<br>°Concentration T-DM1<br>°Concentration DM1<br>°Concentration trastuzumab<br>°ATA<br>°PRO						
	T-DXd	DESTINY-Breast02[28]	ClinicalTrials.gov<br>Pubmed	AstraZeneca	608	°T-DXd<br>°trastuzumab + capecitabine<br>°lapatinib + capecitabine	PFS by BICR	°OS<br>°ORR<br>°DOR<br>°PFS by Investigator	No	No	No	No	NA	NA
	T-DXd	DESTINY-Breast03[29]	ClinicalTrials.gov<br>Pubmed	AstraZeneca	524	°T-DXd<br>°T-DM1	PFS by BICR	°OS<br>°ORR<br>°DOR<br>°PFS by Investigator	No	No	No	No	NA	NA
	T-DXd	DESTINY-Breast04[30]	ClinicalTrials.gov<br>Pubmed	AstraZeneca	557	°T-DXd<br>°TPC (capecitabine, eribulin, gemcitabine, paclitaxel, nab-paclitaxel)	PFS by BICR	°PFS by Investigator<br>°OS<br>°ORR<br>°DOR	Yes	No	No	No	NA	NA
	Sacituzumab-Govitecan	TROPICS02[31]	ClinicalTrials.gov<br>Pubmed	Gilead Sciences	543	°sacituzumab-govitecan<br>°TPC (capecitabine, eribulin, gemcitabine, vinorelbine)	PFS	°ORR<br>°OS<br>°DOR<br>°CBR<br>°EORTC QLQ-C30<br>°TEAE<br>°SAE<br>°Laboratory Abnormalities<br>°ECOG Performance status	No	No	No	No	NA	NA
	Sacituzumab-Govitecan	ASCENT[32]	ClinicalTrials.gov<br>Pubmed	Gilead Sciences	529	°sacituzumab-govitecan<br>°TPC (capecitabine, eribulin, gemcitabine, vinorelbine)	PFS by IRC	°OS<br>°ORR<br>°Time to OR<br>°DOR<br>°TTP<br>°CBR<br>°TEAE<br>°EORTC QLQ-C30	Yes	No	No	No	NA	NA

								°Laboratory Abnormalities						
**Immune checkpoint inhibitors**														
Neoadjuvant	Atezolizumab	Impassion031[33]	ClinicalTrials.gov Pubmed	Roche	333	°chemotherapy + atezolizumab °chemotherapy + placebo	°pCR °pCR in PD-L1 positive tumors	°EFS °EFS in PD-L1 positive tumors °DFS °DFS in PD-L1 positive tumors °OS °OS in PD-L1 positive tumors °EORTC QLQ-C30 °TEAE °Cmin °Cmax °ATA	Yes	No	No	Yes	2.0	No
	Atezolizumab	Impassion050[34]	ClinicalTrials.gov Pubmed	Roche	454	°ddAC + paclitaxel/trastuzumab/pertuzumab + atezolizumab °ddAC + paclitaxel/trastuzumab/pertuzumab + placebo	°pCR in PD-L1 positive tumors °pCR in ITT population	°pCR by HR status °pCR in PD-L1 negative tumors °EFS °DFS °OS °EORTC QLQ-C30 °TEAE °Cmin Atezolizumab °Cmax Atezolizumab °Ctrough for Pertuzumab and Trastuzumab °Cmin T-DM1 °Cmax T-DM1 °ATA Atezolizumab, Trastuzumab, Pertuzumab, T-DM1 °pCR based on PIK3CA mutation °EFS based on PIK3CA mutation °DFS based on PIK3CA mutation	Yes	No	No	No	NA	NA

								°OS based on PIK3CA mutation						
	Atezolizumab	NeoTRIP[35]	ClinicalTrials.gov Pubmed	NA	278	°carboplatin/abraxane + surgery + AC<br>°carboplatin/abraxane + atezolizumab + surgery + AC	EFS	°pCR<br>°COR<br>°DEFS<br>°TEAE	No	Yes	NA	NA	NA	NA
	Pembrolizumab	KEYNOTE522[36]	ClinicalTrials.gov Pubmed	Merck Sharp & Dohme LLC	1174	°chemotherapy + pembrolizumab<br>°chemotherapy + placebo	°pCR<br>°EFS	°EFS in PD-L1 positive tumors<br>°OS<br>°TEAE<br>°EORTC QLQ-C30, EORTC QLQ-BR23	No	No	No	No	NA	NA
Metastatic	Atezolizumab	Impassion130[37]	ClinicalTrials.gov Pubmed	Roche	902	°nab-paclitaxel + atezolizumab<br>°nab-paclitaxel + placebo	°PFS<br>°PFS in PD-L1 positive tumors<br>°OS<br>°OS in PD-L1 positive tumors	°ORR<br>°ORR in PD-L1 positive tumors<br>°DOR<br>°DOR in PD-L1 positive tumors<br>°EORTC QLQ-C30<br>°TEAE<br>°ATA<br>°Cmax<br>°Cmin<br>°Plasma concentrations Paclitaxel	Yes	No	No	No	NA	NA
	Atezolizumab	Impassion131[38]	ClinicalTrials.gov Pubmed	Roche	651	°paclitaxel + atezolizumab<br>°paclitaxel + placebo	°PFS in PD-L1 positive tumors<br>°PFS in ITT population	°OS in PD-L1 positive tumors<br>°OS in ITT population<br>°% alive at 12 and 18m<br>°EORTC QLQ-C30<br>°% Alive without PD at 12m<br>°ORR in PD-L1 positive tumors<br>°ORR<br>°DOR<br>°CBR<br>°Cmin Atezolizumab, Cmin Paclitaxel<br>°Cmax Atezolizumab, Cmax Paclitaxel	Yes	No	No	No	NA	NA

								°TEAE<br>°ATA<br>°OS by PD-L1 status<br>°PFS by PD-L1 status<br>°C-DOR						
	Pembrolizumab	KEYNOTE119[39]	ClinicalTrials.gov<br>Pubmed	Merck Sharp & Dohme LLC	622	°pembrolizumab<br>°chemotherapy (capecitabine, eribulin, gemcitabine, vinorelbine)	°OS in PD-L1 CPS ≥10<br>°OS in PD-L1 CPS ≥1<br>°OS	°ORR in PD-L1 CPS ≥10<br>°ORR in PD-L1 CPS ≥1<br>°ORR<br>°PFS in PD-L1 CPS ≥10<br>°PFS in PD-L1 CPS ≥1<br>°PFS<br>°DOR in PD-L1 CPS ≥10<br>°DOR in PD-L1 CPS ≥1<br>°DOR<br>°DCR in PD-L1 CPS ≥10<br>°DCR in PD-L1 CPS ≥1<br>°DCR<br>°TEAE<br>°Discontinuation due to TEAE	No	No	No	No	NA	NA
	Pembrolizumab	KEYNOTE355[40]	ClinicalTrials.gov<br>Pubmed	Merck Sharp & Dohme LLC	882	°pembrolizumab + nab-paclitaxel<br>°pembrolizumab + paclitaxel<br>°pembrolizumab + gemcitabine/carboplatin<br>°pembrolizumab + chemotherapy<br>°placebo + chemotherapy	°TEAE<br>°Discontinuation due to TEAE<br>°PFS<br>°PFS in PD-L1 CPS ≥1<br>°PFS in PD-L1 CPS ≥10<br>°OS<br>°OS in PD-L1 CPS ≥1<br>°OS in PD-L1 CPS ≥10	°ORR in PD-L1 CPS ≥10<br>°ORR in PD-L1 CPS ≥1<br>°ORR<br>°DOR in PD-L1 CPS ≥10<br>°DOR in PD-L1 CPS ≥1<br>°DOR<br>°DCR in PD-L1 CPS ≥10<br>°DCR in PD-L1 CPS ≥1<br>°DCR<br>°TEAE	Yes	No	No	No	NA	NA

								°Discontinuation due to TEAE<br>°EORTC QLQ-C30, EORTC QLQ-BR23						
**PARP inhibitors**														
Neoadjuvant	Veliparib	BrighTNess[41]	ClinicalTrials.gov<br>Pubmed	AbbVie	634	°veliparib + carboplatin + paclitaxel followed by AC<br>°placebo + carboplatin + paclitaxel followed by AC<br>°placebo + placebo + paclitaxel followed by AC	pCR	°EFS<br>°OS<br>°BCR	No	No	No	No	NA	NA
Adjuvant	Olaparib	OlympiA[42]	ClinicalTrials.gov<br>Pubmed	AstraZeneca	1836	°olaparib<br>°placebo	iDFS	°DDFS<br>°OS<br>°Contralateral Breast Cancers, New Primary Ovarian Cancer, New Primary Fallopian Tube Cancer and New Primary Peritoneal Cancer<br>°FACIT-F, EORTC QLQ-C30	NA	No	No	No	NA	NA
Metastatic	Iniparib	NCT00938652[43]	ClinicalTrials.gov<br>Pubmed	Sanofi	519	°gemcitabine/carboplatin<br>°gemcitabine/carboplatin + iniparib	°PFS<br>°OS	°BOR<br>°ORR	Yes	No	No	No	NA	NA
	Olaparib	OlympiAD[44]	ClinicalTrials.gov<br>Pubmed	AstraZeneca	302	°olaparib<br>°TPC	PFS	°Time to second progression or death<br>°OS<br>°ORR<br>°EORTC QLQ-C30<br>°PFS by BICR	No	No	No	No	NA	NA
	Olaparib	LUCY[45]	ClinicalTrials.gov<br>Pubmed	AstraZeneca	256	olaparib	PFS	°OS<br>°TFST<br>°TSST<br>°TDT<br>°PFS on subsequent therapy<br>°CRR<br>°DOR<br>°TEAE	No	No	No	No	NA	NA
	Talazoparib	EMBRACA[46]	ClinicalTrials.gov<br>Pubmed	Pfizer	431	°talazoparib<br>°TPC	PFS	°ORR<br>°OS	No	No	No	No	NA	NA

								°Cthrough<br>°TEAE<br>°Grade 3/4 Laboratory Abnormalities<br>°Changes in vital signs<br>°Concomitant Medication						
	Veliparib	BROCADE3[47]	ClinicalTrials.gov<br>Pubmed	AbbVie	509	°veliparib + carboplatin + paclitaxel<br>°placebo + carboplatin + paclitaxel	PFS	°OS<br>°CBR<br>°ORR<br>°PFS on subsequent therapy	No	No	No	No	NA	NA
**Tyrosine kinase inhibitors**														
Neoadjuvant	Lapatinib	NSABP protocol B-41[48]	ClinicalTrials.gov<br>Pubmed	GlaxoSmith Kline	529	°trastuzumab<br>°lapatinib	pCR	°clinical complete response<br>°cardiac events<br>°CTCAE side adverse events	No	No	No	No	NA	NA
	Lapatinib	GeparQuinto[49]	ClinicalTrials.gov<br>Pubmed	NA	615	°epirubicine/cyclofosfamide/trastuzumab + docetaxel/trastuzumab<br>°epirubicine/cyclofosfamide/lapatinib + +docetaxel/lapatinib	pCR	°Toxic effects<br>°Compliance<br>°Response rate physical examination<br>°Response rate imaging<br>°Breast conversation rate<br>°pCR according to diff definitions	No	No	No	Yes	2.8	Yes
	Lapatinib	ALLIANCE[50]	ClinicalTrials.gov<br>Pubmed	NA	305	°paclitaxel + trastuzumab<br>°paclitaxel + trastuzumab + lapatinib<br>°paclitaxel + lapatinib	pCR	°pCR axilla<br>°adverse events	No	No	No	No	NA	NA
	Lapatinib	NeoALLTO[51]	ClinicalTrials.gov<br>Pubmed	GlaxoSmith Kline	455	°lapatinib<br>°trastuzumab<br>°lapatinib+trastuzumab	pCR	°locoregional total pCR<br>°objective tumor response rate (physical examination)<br>°Breast conserving rate<br>°Node negativity at surgery	No	No	No	No	NA	NA

								°Safety and tolerability<br>°DFS<br>°OS<br>°Biomarker expression						
	Lapatinib	EPHOS B[52]	ClinicalTrials.gov<br>Pubmed	Novartis	257	°no (neo)adjuvant therapy<br>°trastuzumab<br>°lapatinib<br>°lapatinib + trastuzumab	°Increase in apoptosis<br>°Ki67<br>°RFS	°Angiogenic serum markers<br>°Molecular markers<br>°Time to local recurrence<br>°Time to distant recurrence<br>°OS	No	No	No	Yes	4.0	No
	Pyrotinib	PHEDRA[53]	ClinicalTrials.gov<br>Pubmed	Jiangsu Hengrui Medicine	355	°trastuzumab/docetaxel/pyrotinib<br>°trastuzumab/docetaxel/placebo	pCR	°pCR rate peripheral vs central<br>°Overall response rate<br>°EFS<br>°DFS<br>°DDFS	No	No	No	No	NA	NA
Adjuvant	Lapatinib	TEACH[54]	ClinicalTrials.gov<br>Pubmed	GlaxoSmith Kline	3161	°trastuzumab<br>°lapatinib	DFS	°time to recurrence<br>°OS<br>°Time to CNS recurrence<br>°QoL<br>°Toxic effects	NA	No	No	No	NA	NA
	Lapatinib	ALTTO[55]	ClinicalTrials.gov<br>Pubmed	Novartis	8381	°trastuzumab 52w<br>°lapatinib 52w<br>°trastuzumab + lapatinib 52w<br>°trastuzumab 12 -> lapatinib 34w	DFS	°OS<br>°General Safety<br>°Cardiac Safety<br>°Time to recurrence<br>°Time to distant recurrence<br>°Time to CNS recurrence	NA	No	No	No	NA	NA
	Neratinib	ExteNET[56]	Pubmed	Puma Bio-technology	2840	After 1y trastuzumab<br>°neratinib<br>°placebo	iDFS	°DFS for DCIS<br>°Time to distant recurrence<br>°Distant DFS<br>°Time to CNS recurrence<br>°Overall survival	NA	No	No	No	NA	NA

								°Safety						
Metastatic	Lapatinib	NCT00078572[57]	ClinicalTrials.gov Pubmed	GlaxoSmith Kline	408	°capecitabine<br>°capecitabine + lapatinib	Time to progression	°PFS<br>°OS<br>°clinical benefit rate<br>°Safety	Yes	No	No	No	NA	NA
	Lapatinib	EGF30001[58]	ClinicalTrials.gov Pubmed	GlaxoSmith Kline	579	°paclitaxel + placebo<br>°paclitaxel + lapatinib	Time to progression	°ORR<br>°CBR<br>°Time of response<br>°Event free survival<br>°OS<br>°Safety	Yes	No	No	No	NA	NA
	Lapatinib	EGF104900[59]	ClinicalTrials.gov Pubmed	GlaxoSmith Kline	291	°trastuzumab + lapatinib<br>°lapatinib	PFS	°ORR<br>°CBR<br>°QOL<br>°OS	Yes (exception of bone only disease)	No	No	No	NA	NA
	Lapatinib	NCT00073528[60]	ClinicalTrials.gov Pubmed	Novartis GlaxoSmith Kline	1286	°letrozole + lapatinib<br>°letrozole + placebo	PFS	°ORR<br>°CBR<br>°OS<br>°Safety<br>°PFS	No	No	No	No	NA	NA
	Lapatinib	NCT00281658[61]	ClinicalTrials.gov Pubmed	Novartis	444	°paclitaxel + placebo<br>°paclitaxel + lapatinib	OS 53m	°OS 190m<br>°PFS<br>°ORR<br>°TTR<br>°Safety<br>°CBR<br>°DOR	Yes	No	No	Yes	4.7	No
	Lapatinib	NCIC CTG MA.31[62]	ClinicalTrials.gov Pubmed	Novartis	537	°taxane + lapatinib<br>°taxane + trastuzumab	PFS	°OS<br>°ORR<br>°TTR<br>°DOR<br>°CBR<br>°Adverse Events (AEs)<br>°QoL	No	No	No	No	NA	NA
	Lapatinib	CEREBEL[63]	ClinicalTrials.gov Pubmed	Novartis	540	°capecitabine +trastuzumab<br>°capecitabine + lapatinib	Number of participants with CNS as first site of relapse	°PFS<br>°Time to first CNS progression<br>°Incidence of CNS progression at any time<br>°OS	No	No	No	No	NA	NA

								°ORR<br>°DOR<br>°Safety					
	Lapatinib	ALTERNATIVE[64]	ClinicalTrials.gov<br>Pubmed	Novartis<br>GlaxoSmith<br>Kline	355	°lapatinib + trastuzumab + AI<br>°trastuzumab + AI<br>°lapatinib + AI	PFS<br>(radiologic<br>and non-<br>radiologic<br>response)	°OS<br>°ORR<br>°CBR<br>°Duration of<br>response<br>°QoL	No	No	No	No	NA	NA
	Lapatinib	DETECT III[65]	ClinicalTrials.gov<br>Pubmed	NA	254	°standard therapy<br>°standard therapy + lapatinib	PFS	°ORR<br>°CBR<br>°OS<br>°Dynamic of CTC<br>°QoL<br>°Safety and<br>Tolerability<br>°Intensity of Pain<br>°Level of<br>Compliance	No	No	No	Yes	9.8	No
	Lapatinib	NCT00508274[66]	Pubmed	Novartis	52	°lapatinib + capecitabine	CBR	°PFS<br>°TTR<br>°DOR<br>°CNS as first<br>relapse<br>°Safety	Yes	No	No	No	NA	NA
	Lapatinib	NCT00272987[67]	Pubmed	Novartis	63	°lapatinib (1000mg) + paclitaxel<br>(80mg/m^2)<br>°lapatinib (1000mg) + paclitaxel<br>(70mg/m^2)<br>lapatinib (750mg) + paclitaxel (80mg/m^2)	Safety and<br>tolerability	°CBR<br>°PFS<br>°TTR<br>°DOR	Yes	No	No	No	NA	NA
	Lapatinib	CALGB 40302[68]	Pubmed	NA	295	°fulvestrant + lapatinib<br>°fulvestrant + placebo	PFS	°Toxicity<br>°Objective tumor<br>response<br>°OS	Yes<br>(exception<br>of bone<br>only<br>disease)	No	No	No	NA	NA
	Neratinib	NALA[69]	Pubmed	Puma Bio-<br>technology	621	°capecitabine + neratinib<br>°capecitabine + lapatinib	°PFS<br>°OS	°Time to<br>intervention for<br>CNS<br>°ORR<br>°Investigator<br>assessed PFS<br>°CBR<br>°DOR<br>°HRQoL<br>°Safety	Yes	No	No	No	NA	NA

	Pyrotinib	PHOEBE[70]	ClinicalTrials.gov<br>Pubmed	Jiangsu HengRui Medicine	267	°capecitabine + pyrotinib<br>°capecitabine + lapatinib	PFS	°Safety<br>°OS<br>°ORR<br>°TTP<br>°DOR<br>°CBR	Yes	No	No	No	NA	NA
	Pyrotinib	Phenix[71]	Clinicaltrials.gov	Jiangsu HengRui Medicine	279	°capecitabine + pyrotinib<br>°capecitabine + placebo	PFS by IRC	°PFS by investigator<br>°ORR<br>°Disease control rate<br>°CBR<br>°DOR<br>°OS<br>°Safety	Yes	No	No	No	NA	NA
**mTOR inhibitors**														
Neoadjuvant	Everolimus	GeparQuinto[72]	ClinicalTrials.gov<br>Pubmed	NA	1948	°epirubicine/cyclofosfamide/trastuzumab + docetaxel/trastuzumab<br>°epirubicine/cyclofosfamide/lapatinib + +docetaxel/lapatinib<br>°paclitaxel<br>°epirubicine/cyclofosfamide + docetaxel<br>°epirubicine/cyclofosfamide + docetaxel + bevacuzimab<br>°paclitaxel + everolimus	pCR	°DFS<br>°LRFS<br>°LRRFS<br>°RRFS<br>°DDFS<br>°Cerebral DFS<br>°OS	No	No	No	Yes	10.8	Yes
Adjuvant	Everolimus	NCT01805271[73]	ClinicalTrials.gov<br>Pubmed	NA	1278	°endocrine therapy + everolimus<br>°endocrine therapy + placebo	DFS	°OS<br>°EFS<br>°Distant metastases - FS<br>°Second malignancies<br>°Toxicity	NA	No	No	No	NA	NA
	Everolimus	MAINtenance Afinitor[74]	ClinicalTrials.gov<br>Pubmed	NA	110	°endocrine therapy + everolimus<br>°endocrine therapy + placebo	PFS	°ORR<br>°OS<br>°Safety	NA	No	No	Yes	16.3	No
Metastatic	Everolimus	4EVER[75]	ClinicalTrials.gov<br>Pubmed	Novartis	281	°everolimus + exemestane<br>°exemestane	ORR	°PFS<br>°ORR<br>°OS<br>°Safety<br>°Resource utilization<br>°HRQoL	Yes (exception of bone only disease)	No	No	No	NA	NA
	Everolimus	Everexes[76]	ClinicalTrials.gov	Novartis	235	everolimus + exemestane	Safety<br>Tolerability	°ORR<br>°CBR<br>°TTD	Yes (exception of bone	No	No	No	NA	NA

									only disease)					
	Everolimus	Bolero-1[77]	ClinicalTrials.gov Pubmed	Novartis	719	°trastuzumab + paclitaxel + everolimus °trastuzumab + paclitaxel + placebo	PFS	°OS °objective response rate °CBR °Safety	Yes (exception of bone only disease)	No	No	No	NA	NA
	Everolimus	Bolero-2[78]	ClinicalTrials.gov Pubmed	Novartis	724	°everolimus + exemestane °exemestane	PFS	°ORR °CBR °OS °QoL °Safety °changes in bone marker levels	No	No	No	No	NA	NA
	Everolimus	Bolero-3[79]	ClinicalTrials.gov Pubmed	Novartis	569	°trastuzumab + vinolrebine + everolimus °trastuzumab + vinolrebine + placebo	PFS	°OS °objective response rate °CBR °Safety	No	No	No	No	NA	NA
	Everolimus	BALLET[80]	Pubmed	Novartis	1151	°everolimus + exemestane	Safety	°Grade 3-4 AE	No	No	No	No	NA	NA
	Everolimus	SWOG 1222[81]	ClinicalTrials.gov Pubmed	AstraZeneca Novartis	37	°fulvestrant + placebo + placebo °fulvestrant + everolimus + placebo °fulvestrant + everolimus + anastrazole	PFS	°OS °Adverse Events °CTC analyses	No	No	No	No	NA	NA
	Everolimus	INPRES[82]	ClinicalTrials.gov	NA	44	°exemestane + everolimus	Everolimus AUC	°Correlation early metabolic response and PFS °Correlation early metabolic response and AUC °Effect dose escalation on metabolic response °Correlation AUC and frequency of adverse events	No	No	No	Yes	26.0	Yes
	Everolimus	IMPROVE[83]	Pubmed	NA	77	°exemestane + everolimus °capecitabine + bevacizumab	Patient preference for type of therapy	°PFS °OS °treatment satisfaction °Qol °Safety	No	No	No	Yes	24.7	No
	Temsirolimus	NCT00083993[84]	ClinicalTrials.gov Pubmed	Pfizer	1112	°letrozole + placebo °letrozole + temsirolimus	PFS	°OS °Tumor response °Clinical benefit	Yes	No	No	No	NA	NA

								°TTP<br>°DOR<br>°TTF<br>°Safety<br>°QoL						
**PI3K/AKT pathway inhibitors**														
Metastatic	Alpelisib	SOLAR1[85]	ClinicalTrials.gov	Novartis	572	°fulvestrant + alpelisib<br>°fulvestrant + placebo	PFS	°OS<br>°Subgroup analyses PIK3CA<br>°PFS according to ctDNA<br>°Overall response<br>°CBR<br>°Safety	Yes (exception of bone only disease)	No	No	No	NA	NA
	Buparlisib	BELLE2[86]	Pubmed	Novartis	1147	°fulvestrant + buparlisib<br>°fulvestrant + placebo	PFS	°OS<br>°CBR<br>°ORR<br>°PK<br>°Safety<br>°QoL	No	No	No	Yes	13.0	No
	Buparlisib	BELLE3[87]	ClinicalTrials.gov<br>Pubmed	Novartis	432	°fulvestrant + buparlisib<br>°fulvestrant + placebo	PFS by investigator	°OS<br>°PFS by PIK3CA<br>°OS by PIK3CA<br>°ORR by PIK3CA<br>°CBR by PIK3CA<br>°Safety and Tolerability<br>°PK<br>°HRQoL<br>°Time to Definitive Deterioration of ECOG Performance Status	Yes	No	No	No	NA	NA
	Buparlisib	BELLE4[88]	ClinicalTrials.gov	Novartis	416	°paclitaxel + buparlisib<br>°paclitaxel + placebo	°PFS<br>°PFS by PIK3CA	°OS<br>°ORR<br>°CBR<br>°PK<br>°Safety	No	No	No	No	NA	NA
	Ipatasertib	IPATunity130[89]	ClinicalTrials.gov	Roche	222	°paclitaxel + ipatasertib<br>°paclitaxel + placebo	PFS	°OS<br>°ORR<br>°DOR<br>°CBR<br>°Safety	Yes	No	No	No	NA	NA

								°Patient reported Outcomes						
	Taselisib	SANDPIPER[90]	ClinicalTrials.gov	Roche	631	°fulvestrant + taselisib<br>°fulvestrant + placebo	PFS by investigator	°OS<br>°CBR<br>°Duration of objective response<br>°BICR-PFS<br>°TTD<br>°HRQoL<br>°Safety	No	No	No	No	NA	NA
	Capivasertib	Capitello 291[91]	ClinicalTrials.gov<br>Pubmed	AstraZeneca	708	°fulvestrant + capivasertib<br>°fulvestrant + placebo	PFS by investigator	°OS<br>°ORR<br>°Safety	Yes (exception of bone only disease)	No	No	No	NA	NA
**Others**														
Metastatic	Tucidinostat	ACE[92]	ClinicalTrials.gov<br>Pubmed	Chipscreen Biosciences	365	°tucidinistat+ exemestane<br>°placebo + exemestane	°PFS<br>°PK Chidamide, PK Exemestan e<br>°Acetylatio n level histone H3	°OS<br>°DOR<br>°ORR<br>°CBR	Yes	No	No	No	NA	NA
	Margetuximab	SOPHIA[93]	ClinicalTrials.gov<br>Pubmed	Macro-Genics	536	°margetuximab + chemotherapy<br>°trastuzumab + exemestane	°PFS<br>°OS<br>°Grade 3 CTCEA	°ORR<br>°Safety	No	No	No	No	NA	NA

AC: anthracycline; AE: adverse events; AEPI: Adverse Events of Primary Interest; AESI: Adverse Events of Special Interest; AI: aromatase inhibition; ATA: anti-therapeutic antibodies; AUC: area under the curve; BCS: breast conserving surgery; BICR: Blinded independent central reviews; BOR: best overall response rate; CBR: clinical benefit rate; C-DOR: duration of confirmed response; Cmax: maximum concentration; Cmin: minimum concentration; CNS: central nervous system; CPS: combined positive score; Ctrough: trough concentration; CRR: clinical response rate; CTC: circulating tumor cell; CTCEA: Common Terminology Criteria for Adverse Events; ctDNA: circulating tumor DNA; DCIS: ductal carcinoma in situ; DCR: disease control rate; ddAC: dose-dense anthracycline; DDFS: distant disease free survival; DEFS: distant event free survival; DFS: disease free survival; DM1: emtansine; DOR: duration of response; DRFI: distant recurrence free interval; DRFS: distant recurrence free survival; EFS: event free survival; ECOG: Eastern Cooperative Oncology Group; EORTC QLQ-BM22: The European Organisation for Research and Treatment of Cancer Quality of Life Questionnaire for patients with bone metastases; EORTC QLQ-BR23: The European Organization for Research and Treatment of Cancer Breast Cancer-Specific Quality of Life Questionnaire; EORTC QLQ-C30: European Organization for Research and Treatment of Cancer Quality of Life Questionnaire Version 3.0; EQ-5D-5L: Euro Quality of Life 5 dimensions 5 level; ER: estrogen receptor; FACIT-F: Functional Assessment of Chronic Illness Therapy – Fatigue; FACT-B: Functional Assessment of Cancer Therapy – Breast; FACT-C: Functional Assessment of Cancer Therapy Colorectal Cancel Module; FACT-TaxS: Functional Assessment of Cancer Therapy Taxane Score; HR: hormone receptor; HRQOL: Health Related Quality of Life; iDFS: invasive disease free survival; ILC: invasive lobular carcinoma; IRC: independent review committee; ITT: intention to treat; NA: not applicable; LRFS: local recurrence free survival; LRRFS: locoregional recurrence free survival; LVEF: left ventricular ejection fraction; mBPI-sf: modified Brief Pain Inventory-short form; OR: objective response; ORR: overall response rate; OS: overall survival; pCR: pathological complete response; PD: progressive disease; PFS: progression free survival; PK: pharmacokinetics; QALY: quality adjusted life year; QOL: quality of life; QTc: QT duration corrected for heart rate; RFS: recurrence free survival; RSCL: Rotterdam Symptom Checklist; SAE: serious adverse events; SERD: selective estrogen receptor degrader; SOC: standard of care; T-DM1: trastuzumab-emtansine; TDT: Time to Study Treatment Discontinuation or Death; T-DXd: trastuzumab-deruxtecan; TEAE: treatment emergent adverse event; TFST: time to first subsequent therapy; TMB: tumour mutational burden; TPC: treatment of physician’s choice; TSST: time to second subsequent therapy; TTD: time to deterioration; TTF: time to treatment failure; TTP: time to progression; TTR: time to response; WPAI: Work Productivity and Activity Impairment

References

1. Alsaleh, K. *et al.* Neoadjuvant endocrine therapy with or without palbociclib in low-risk patients: a phase III randomized double-blind SAFIA trial. *J Cancer Res Clin Oncol* 1 (2023) doi:10.1007/S00432-023-04588-3.
2. Harbeck, N. *et al.* Adjuvant abemaciclib combined with endocrine therapy for high-risk early breast cancer: updated efficacy and Ki-67 analysis from the monarchE study. *Annals of Oncology* **32**, 1571–1581 (2021).
3. Loibl, S. *et al.* Palbociclib for residual high-risk invasive HR-positive and HER2-negative early breast cancer-the penelope-B trial. *Journal of Clinical Oncology* **39**, 1518–1530 (2021).
4. Gnant, M. *et al.* Adjuvant Palbociclib for Early Breast Cancer: The PALLAS Trial Results (ABCSG-42/AFT-05/BIG-14-03). *J Clin Oncol* **40**, 282–293 (2022).
5. Sledge, G. W. *et al.* MONARCH 2: Abemaciclib in combination with fulvestrant in women with HR+/HER2-advanced breast cancer who had progressed while receiving endocrine therapy. *Journal of Clinical Oncology* **35**, 2875–2884 (2017).
6. Johnston, S. *et al.* MONARCH 3 final PFS: a randomized study of abemaciclib as initial therapy for advanced breast cancer. *NPJ Breast Cancer* **5**, (2019).
7. Zhang, Q. Y. *et al.* MONARCH plus: abemaciclib plus endocrine therapy in women with HR+/HER2– advanced breast cancer: the multinational randomized phase III study. *Ther Adv Med Oncol* **12**, (2020).
8. Finn, R. S. *et al.* Palbociclib and Letrozole in Advanced Breast Cancer. *New England Journal of Medicine* **375**, 1925–1936 (2016).
9. Cristofanilli, M. *et al.* Fulvestrant plus palbociclib versus fulvestrant plus placebo for treatment of hormone-receptor-positive, HER2-negative metastatic breast cancer that progressed on previous endocrine therapy (PALOMA-3): final analysis of the multicentre, double-blind, phase 3 randomised controlled trial. *Lancet Oncol* **17**, 425–439 (2016).
10. Xu, B. *et al.* Palbociclib plus letrozole versus placebo plus letrozole in Asian postmenopausal women with oestrogen receptor–positive/human epidermal growth factor receptor 2–negative advanced breast cancer: Primary results from PALOMA-4. *Eur J Cancer* **175**, 236–245 (2022).
11. Bidard, F. C. *et al.* Switch to fulvestrant and palbociclib versus no switch in advanced breast cancer with rising ESR1 mutation during aromatase inhibitor and palbociclib therapy (PADA-1): a randomised, open-label, multicentre, phase 3 trial. *Lancet Oncol* **23**, 1367–1377 (2022).
12. Martín, M. *et al.* Overall survival with palbociclib plus endocrine therapy versus capecitabine in postmenopausal patients with hormone receptor-positive, HER2-negative metastatic breast cancer in the PEARL study. *Eur J Cancer* **168**, 12–24 (2022).
13. Hortobagyi, G. N. *et al.* Updated results from MONALEESA-2, a phase III trial of first-line ribociclib plus letrozole versus placebo plus letrozole in hormone receptor-positive, HER2-negative advanced breast cancer. *Annals of Oncology* **29**, 1541–1547 (2018).
14. Slamon, D. J. *et al.* Phase III randomized study of ribociclib and fulvestrant in hormone receptor-positive, human epidermal growth factor receptor 2-negative advanced breast cancer: MONALEESA-3. *Journal of Clinical Oncology* **36**, 2465–2472 (2018).
15. Tripathy, D. *et al.* Ribociclib plus endocrine therapy for premenopausal women with hormone-receptor-positive, advanced breast cancer (MONALEESA-7): a randomised phase 3 trial. *Lancet Oncol* **19**, 904–915 (2018).
16. Peuker, C. A. *et al.* Treatment with ribociclib shows favourable immunomodulatory effects in patients with hormone receptor-positive breast cancer—findings from the RIBECCA trial. *Eur J Cancer* **162**, 45–55 (2022).
17. De Laurentiis, M. *et al.* Full population results from the core phase of CompLEEment-1, a phase 3b study of ribociclib plus letrozole as first-line therapy for advanced breast cancer in an expanded population. *Breast Cancer Res Treat* **189**, 689 (2021).
18. Malorni, L. *et al.* Serum thymidine kinase activity in patients with HR-positive/HER2-negative advanced breast cancer treated with ribociclib plus letrozole: Results from the prospective BioItaLEE trial. *Eur J Cancer* **186**, 1–11 (2023).
19. Bidard, F. C. *et al.* Elacestrant (oral selective estrogen receptor degrader) Versus Standard Endocrine Therapy for Estrogen Receptor–Positive, Human Epidermal Growth Factor Receptor 2–Negative Advanced Breast Cancer: Results From the Randomized Phase III EMERALD Trial. *Journal of Clinical Oncology* **40**, 3246 (2022).
20. Hurvitz, S. A. *et al.* Neoadjuvant trastuzumab, pertuzumab, and chemotherapy versus trastuzumab emtansine plus pertuzumab in patients with HER2-positive breast cancer (KRISTINE): a randomised, open-label, multicentre, phase 3 trial. *Lancet Oncol* **19**, 115–126 (2018).
21. Krop, I. E. *et al.* Trastuzumab Emtansine Plus Pertuzumab Versus Taxane Plus Trastuzumab Plus Pertuzumab After Anthracycline for High-Risk Human Epidermal Growth Factor Receptor 2–Positive Early Breast Cancer: The Phase III KAITLIN Study. *Journal of Clinical Oncology* **40**, 438 (2022).
22. von Minckwitz, G. *et al.* Trastuzumab Emtansine for Residual Invasive HER2-Positive Breast Cancer. *New England Journal of Medicine* **380**, 617–628 (2019).
23. Diéras, V. *et al.* Trastuzumab emtansine versus capecitabine plus lapatinib in patients with previously treated HER2-positive advanced breast cancer (EMILIA): a descriptive analysis of final overall survival results from a randomised, open-label, phase 3 trial. *Lancet Oncol* **18**, 732 (2017).

24. Krop, I. E. *et al.* Trastuzumab emtansine versus treatment of physician's choice in patients with previously treated HER2-positive metastatic breast cancer (TH3RESA): final overall survival results from a randomised open-label phase 3 trial. *Lancet Oncol* **18**, 743–754 (2017).
25. Wuerstlein, R. *et al.* Final results of the global and Asia cohorts of KAMILLA, a phase IIIB safety trial of trastuzumab emtansine in patients with HER2-positive advanced breast cancer. *ESMO Open* **7**, 100561 (2022).
26. Perez, E. A. *et al.* Trastuzumab emtansine with or without pertuzumab versus trastuzumab with taxane for human epidermal growth factor receptor 2–positive advanced breast cancer: Final results from MARIANNE. *Cancer* **125**, 3974–3984 (2019).
27. Wang, X. *et al.* Primary results of ELAINA: a randomized, multicenter, open-label, phase III study of the efficacy and safety of trastuzumab emtansine vs . lapatinib plus capecitabine in Chinese patients with HER2-positive locally advanced or metastatic breast cancer who have received prior trastuzumab-based therapy. *Translational Breast Cancer Research* **4**, 3–3 (2023).
28. André, F. *et al.* Trastuzumab deruxtecan versus treatment of physician's choice in patients with HER2-positive metastatic breast cancer (DESTINY-Breast02): a randomised, open-label, multicentre, phase 3 trial. *The Lancet* **401**, 1773–1785 (2023).
29. Cortés, J. *et al.* Trastuzumab Deruxtecan versus Trastuzumab Emtansine for Breast Cancer. *New England Journal of Medicine* **386**, 1143–1154 (2022).
30. Modi, S. *et al.* Trastuzumab Deruxtecan in Previously Treated HER2-Low Advanced Breast Cancer. *New England Journal of Medicine* **387**, 9–20 (2022).
31. Rugo, H. S. *et al.* Sacituzumab Govitecan in Hormone Receptor-Positive/Human Epidermal Growth Factor Receptor 2-Negative Metastatic Breast Cancer. *Journal of Clinical Oncology* **40**, 3365–3376 (2022).
32. Bardia, A. *et al.* Sacituzumab Govitecan in Metastatic Triple-Negative Breast Cancer. *New England Journal of Medicine* **384**, 1529–1541 (2021).
33. Mittendorf, E. A. *et al.* Neoadjuvant atezolizumab in combination with sequential nab-paclitaxel and anthracycline-based chemotherapy versus placebo and chemotherapy in patients with early-stage triple-negative breast cancer (IMpassion031): a randomised, double-blind, phase 3 trial. *The Lancet* **396**, 1090–1100 (2020).
34. Huober, J. *et al.* Atezolizumab With Neoadjuvant Anti–Human Epidermal Growth Factor Receptor 2 Therapy and Chemotherapy in Human Epidermal Growth Factor Receptor 2–Positive Early Breast Cancer: Primary Results of the Randomized Phase III IMpassion050 Trial. *Journal of Clinical Oncology* **40**, 2946 (2022).
35. Gianni, L. *et al.* Pathologic complete response (pCR) to neoadjuvant treatment with or without atezolizumab in triple-negative, early high-risk and locally advanced breast cancer: NeoTRIP Michelangelo randomized study. *Annals of Oncology* **33**, 534–543 (2022).
36. Schmid, P. *et al.* Event-free Survival with Pembrolizumab in Early Triple-Negative Breast Cancer. *New England Journal of Medicine* **386**, 556–567 (2022).
37. Schmid, P. *et al.* Atezolizumab and Nab-Paclitaxel in Advanced Triple-Negative Breast Cancer. *New England Journal of Medicine* **379**, 2108–2121 (2018).
38. Miles, D. *et al.* Primary results from IMpassion131, a double-blind, placebo-controlled, randomised phase III trial of first-line paclitaxel with or without atezolizumab for unresectable locally advanced/metastatic triple-negative breast cancer. *Annals of Oncology* **32**, 994–1004 (2021).
39. Winer, E. P. *et al.* Pembrolizumab versus investigator-choice chemotherapy for metastatic triple-negative breast cancer (KEYNOTE-119): a randomised, open-label, phase 3 trial. *Lancet Oncol* **22**, 499–511 (2021).
40. Cortes, J. *et al.* Pembrolizumab plus Chemotherapy in Advanced Triple-Negative Breast Cancer. *New England Journal of Medicine* **387**, 217–226 (2022).
41. Loibl, S. *et al.* Addition of the PARP inhibitor veliparib plus carboplatin or carboplatin alone to standard neoadjuvant chemotherapy in triple-negative breast cancer (BrighTNess): a randomised, phase 3 trial. *Lancet Oncol* **19**, 497–509 (2018).
42. Tutt, A. N. J. *et al.* Adjuvant Olaparib for Patients with BRCA1- or BRCA2-Mutated Breast Cancer. *N Engl J Med* **384**, 2394 (2021).
43. O'Shaughnessy, J. *et al.* Phase III study of iniparib plus gemcitabine and carboplatin versus gemcitabine and carboplatin in patients with metastatic triple-negative breast cancer. *Journal of Clinical Oncology* **32**, 3840–3847 (2014).
44. Robson, M. *et al.* Olaparib for Metastatic Breast Cancer in Patients with a Germline BRCA Mutation . *New England Journal of Medicine* **377**, 523–533 (2017).
45. Gelmon, K. A. *et al.* Clinical effectiveness of olaparib monotherapy in germline BRCA-mutated, HER2-negative metastatic breast cancer in a real-world setting: phase IIIb LUCY interim analysis. *Eur J Cancer* **152**, 68–77 (2021).
46. Litton, J. K. *et al.* Talazoparib in Patients with Advanced Breast Cancer and a Germline BRCA Mutation . *New England Journal of Medicine* **379**, 753–763 (2018).
47. Diéras, V. *et al.* Veliparib with carboplatin and paclitaxel in BRCA-mutated advanced breast cancer (BROCADE3): a randomised, double-blind, placebo-controlled, phase 3 trial. *Lancet Oncol* **21**, 1269–1282 (2020).
48. Robidoux, A. *et al.* Lapatinib as a component of neoadjuvant therapy for HER2-positive operable breast cancer (NSABP protocol B-41): an open-label, randomised phase 3 trial. *Lancet Oncol* **14**, 1183–1192 (2013).
49. Untch, M. *et al.* Lapatinib versus trastuzumab in combination with neoadjuvant anthracycline-taxane-based chemotherapy (GeparQuinto, GBG 44): a randomised phase 3 trial. *Lancet Oncol* **13**, 135–144 (2012).

50. Carey, L. A. *et al.* Molecular Heterogeneity and Response to Neoadjuvant Human Epidermal Growth Factor Receptor 2 Targeting in CALGB 40601, a Randomized Phase III Trial of Paclitaxel Plus Trastuzumab With or Without Lapatinib. *Journal of Clinical Oncology* **34**, 542 (2016).
51. Baselga, J. *et al.* Lapatinib with trastuzumab for HER2-positive early breast cancer (NeoALTTO): a randomised, open-label, multicentre, phase 3 trial. *Lancet* **379**, 633 (2012).
52. Bundred, N. *et al.* Combined Perioperative Lapatinib and Trastuzumab in Early HER2-Positive Breast Cancer Identifies Early Responders: Randomized UK EPHOS-B Trial Long-Term Results. *Clinical Cancer Research* **28**, 1323 (2022).
53. Wu, J. *et al.* Neoadjuvant pyrotinib, trastuzumab, and docetaxel for HER2-positive breast cancer (PHEDRA): a double-blind, randomized phase 3 trial. *BMC Med* **20**, (2022).
54. Goss, P. E. *et al.* Adjuvant lapatinib for women with early-stage HER2-positive breast cancer: a randomised, controlled, phase 3 trial. *Lancet Oncol* **14**, 88–96 (2013).
55. Piccart-Gebhart, M. *et al.* Adjuvant Lapatinib and Trastuzumab for Early Human Epidermal Growth Factor Receptor 2–Positive Breast Cancer: Results From the Randomized Phase III Adjuvant Lapatinib and/or Trastuzumab Treatment Optimization Trial. *Journal of Clinical Oncology* **34**, 1034 (2016).
56. Chan, A. *et al.* Final Efficacy Results of Neratinib in HER2-positive Hormone Receptor-positive Early-stage Breast Cancer From the Phase III ExteNET Trial. *Clin Breast Cancer* **21**, 80-91.e7 (2021).
57. Geyer, C. E., Forster, J. & Lindquist, D. Lapatinib plus capecitabine for HER2-positive advanced breast cancer. *Advances in Breast Cancer* **5**, 45 (2008).
58. Di Leo, A. *et al.* Phase III, double-blind, randomized study comparing lapatinib plus paclitaxel with placebo plus paclitaxel as first-line treatment for metastatic breast cancer. *J Clin Oncol* **26**, 5544–52 (2008).
59. Blackwell, K. L. *et al.* Overall survival benefit with lapatinib in combination with trastuzumab for patients with human epidermal growth factor receptor 2-positive metastatic breast cancer: Final results from the EGF104900 study. *Journal of Clinical Oncology* **30**, 2585–2592 (2012).
60. Johnston, S. *et al.* Lapatinib combined with letrozole versus letrozole and placebo as first-line therapy for postmenopausal hormone receptor - Positive metastatic breast cancer. *Journal of Clinical Oncology* **27**, 5538–5546 (2009).
61. Guan, Z. *et al.* Randomized trial of lapatinib versus placebo added to paclitaxel in the treatment of human epidermal growth factor receptor 2-overexpressing metastatic breast cancer. *J Clin Oncol* **31**, 1947–1953 (2013).
62. Gelmon, K. A. *et al.* Lapatinib or trastuzumab plus taxane therapy for human epidermal growth factor receptor 2-positive advanced breast cancer: Final results of NCIC CTG MA.31. *Journal of Clinical Oncology* **33**, 1574–1583 (2015).
63. Pivot, X. *et al.* CEREBEL (EGF111438): A phase III, randomized, open-label study of lapatinib plus capecitabine versus trastuzumab plus capecitabine in patients with human epidermal growth factor receptor 2-positive metastatic breast cancer. *Journal of Clinical Oncology* **33**, 1564–1573 (2015).
64. Johnston, S. R. D. *et al.* Phase III, randomized study of dual human epidermal growth factor receptor 2 (HER2) blockade with lapatinib plus trastuzumab in combination with an aromatase inhibitor in postmenopausal women with HER2-positive, hormone receptor–positive metastatic breast cancer: Updated results of alternative. *Journal of Clinical Oncology* **39**, 79–89 (2021).
65. Fehm, T. *et al.* HER2 status of circulating tumor cells in patients with metastatic breast cancer: A prospective, multicenter trial. *Breast Cancer Res Treat* **124**, 403–412 (2010).
66. Xu, B. H. *et al.* Lapatinib plus capecitabine in treating HER2-positive advanced breast cancer: efficacy, safety, and biomarker results from Chinese patients. *Chin J Cancer* **30**, 327 (2011).
67. Esteva, F. J. *et al.* An Open-Label Safety Study of Lapatinib Plus Trastuzumab Plus Paclitaxel in First-Line HER2-Positive Metastatic Breast Cancer. *Oncologist* **18**, 661 (2013).
68. Burstein, H. J. *et al.* Endocrine Therapy With or Without Inhibition of Epidermal Growth Factor Receptor and Human Epidermal Growth Factor Receptor 2: A Randomized, Double-Blind, Placebo-Controlled Phase III Trial of Fulvestrant With or Without Lapatinib for Postmenopausal Women With Hormone Receptor–Positive Advanced Breast Cancer—CALGB 40302 (Alliance). *Journal of Clinical Oncology* **32**, 3959 (2014).
69. Saura, C. *et al.* Neratinib plus capecitabine versus lapatinib plus capecitabine in HER2-positive metastatic breast cancer previously treated with ≥ 2 HER2-directed regimens: Phase III NALA trial. *Journal of Clinical Oncology* **38**, 3138–3149 (2020).
70. Xu, B. *et al.* Pyrotinib plus capecitabine versus lapatinib plus capecitabine for the treatment of HER2-positive metastatic breast cancer (PHOEBE): a multicentre, open-label, randomised, controlled, phase 3 trial. *Lancet Oncol* **22**, 351–360 (2021).
71. Yan, M. *et al.* Pyrotinib plus capecitabine for human epidermal factor receptor 2-positive metastatic breast cancer after trastuzumab and taxanes (PHENIX): a randomized, double-blind, placebo-controlled phase 3 study. *Translational Breast Cancer Research* **1**, 13–13 (2020).
72. von Minckwitz, G. *et al.* Survival after neoadjuvant chemotherapy with or without bevacizumab or everolimus for HER2-negative primary breast cancer (GBG 44-GeparQuinto). *Annals of Oncology* **25**, 2363–2372 (2014).
73. Bachelot, T. *et al.* Everolimus Added to Adjuvant Endocrine Therapy in Patients With High-Risk Hormone Receptor-Positive, Human Epidermal Growth Factor Receptor 2-Negative Primary Breast Cancer. *Journal of Clinical Oncology* **386**, (2022).

74. Guarneri, V. *et al.* Everolimus plus aromatase inhibitors as maintenance therapy after first-line chemotherapy: Final results of the phase III randomised MAIN-A (MAINtenance Afinitor) trial. *Eur J Cancer* **154**, 21–29 (2021).
75. Tesch, H. *et al.* Efficacy and safety of everolimus plus exemestane in postmenopausal women with hormone receptor-positive, human epidermal growth factor receptor 2-negative locally advanced or metastatic breast cancer: Results of the single-arm, phase IIIB 4EVER trial. *Int J Cancer* **144**, 877 (2019).
76. Im, Y. H. *et al.* Safety and efficacy of everolimus (EVE) plus exemestane (EXE) in postmenopausal women with locally advanced or metastatic breast cancer: final results from EVEREXES. *Breast Cancer Res Treat* **188**, 77–89 (2021).
77. Hurvitz, S. A. *et al.* Combination of everolimus with trastuzumab plus paclitaxel as first-line treatment for patients with HER2-positive advanced breast cancer (BOLERO-1): a phase 3, randomised, double-blind, multicentre trial. *Lancet Oncol* **16**, 816–829 (2015).
78. Yardley, D. A. *et al.* Everolimus Plus Exemestane in Postmenopausal Patients with HR+ Breast Cancer: BOLERO-2 Final Progression-Free Survival Analysis. *Adv Ther* **30**, 870 (2013).
79. André, F. *et al.* Everolimus for women with trastuzumab-resistant, HER2-positive, advanced breast cancer (BOLERO-3): a randomised, double-blind, placebo-controlled phase 3 trial. *Lancet Oncol* **15**, 580–591 (2014).
80. Jerusalem, G. *et al.* Safety of everolimus plus exemestane in patients with hormone-receptor–positive, HER2–negative locally advanced or metastatic breast cancer progressing on prior non-steroidal aromatase inhibitors: primary results of a phase IIIb, open-label, single-arm, expanded-access multicenter trial (BALLET). *Annals of Oncology* **27**, 1719–1725 (2016).
81. Moore, H. C. F. *et al.* A randomized trial of fulvestrant, everolimus and anastrozole in the front-line treatment of advanced hormone receptor-positive breast cancer, SWOG S1222. *Clin Cancer Res* **28**, 611 (2022).
82. Willemsen, A. E. C. A. B. *et al.* Everolimus Exposure and Early Metabolic Response as Predictors of Treatment Outcomes in Breast Cancer Patients Treated with Everolimus and Exemestane. *Target Oncol* **13**, 641–648 (2018).
83. Decker, T. *et al.* Final results from IMPROVE: a randomized, controlled, open-label, two-arm, cross-over phase IV study to determine patients' preference for everolimus in combination with exemestane or capecitabine in combination with bevacizumab in advanced HR-positive, HER2-negative breast cancer. *BMC Cancer* **20**, (2020).
84. Wolff, A. C. *et al.* Randomized Phase III Placebo-Controlled Trial of Letrozole Plus Oral Temsirolimus As First-Line Endocrine Therapy in Postmenopausal Women With Locally Advanced or Metastatic Breast Cancer. *Journal of Clinical Oncology* **31**, 195 (2013).
85. André, F. *et al.* Alpelisib for PIK3CA -Mutated, Hormone Receptor–Positive Advanced Breast Cancer . *New England Journal of Medicine* **380**, 1929–1940 (2019).
86. Baselga, J. *et al.* Buparlisib plus fulvestrant versus placebo plus fulvestrant in postmenopausal, hormone receptor-positive, HER2-negative, advanced breast cancer (BELLE-2): a randomised, double-blind, placebo-controlled, phase 3 trial. *Lancet Oncol* **18**, 904 (2017).
87. Di Leo, A. *et al.* Buparlisib plus fulvestrant in postmenopausal women with hormone-receptor-positive, HER2-negative, advanced breast cancer progressing on or after mTOR inhibition (BELLE-3): a randomised, double-blind, placebo-controlled, phase 3 trial. *Lancet Oncol* **19**, 87–100 (2018).
88. Martín, M. *et al.* A randomized adaptive phase II/III study of buparlisib, a pan-class I PI3K inhibitor, combined with paclitaxel for the treatment of HER2- advanced breast cancer (BELLE-4). *Annals of Oncology* **28**, 313–320 (2017).
89. Turner, N. *et al.* Ipatasertib plus paclitaxel for PIK3CA/AKT1/PTEN-altered hormone receptor-positive HER2-negative advanced breast cancer: primary results from cohort B of the IPATunity130 randomized phase 3 trial. *Breast Cancer Res Treat* **191**, 565 (2022).
90. Dent, S. *et al.* Phase III randomized study of taselisib or placebo with fulvestrant in estrogen receptor-positive, PIK3CA-mutant, HER2-negative, advanced breast cancer: the SANDPIPER trial. *Ann Oncol* **32**, 197 (2021).
91. Turner, N. C. *et al.* Capivasertib in Hormone Receptor–Positive Advanced Breast Cancer. *New England Journal of Medicine* **388**, 2058–2070 (2023).
92. Jiang, Z. *et al.* Tucidinostat plus exemestane for postmenopausal patients with advanced, hormone receptor-positive breast cancer (ACE): a randomised, double-blind, placebo-controlled, phase 3 trial. *Lancet Oncol* **20**, 806–815 (2019).
93. Rugo, H. S. *et al.* Margetuximab Versus Trastuzumab in Patients With Previously Treated HER2-Positive Advanced Breast Cancer (SOPHIA): Final Overall Survival Results From a Randomized Phase 3 Trial. *Journal of Clinical Oncology* **41**, 198 (2023).
